# Metaplasticity and behavior: how training and inflammation affect plastic potential within the spinal cord and recovery after injury

**DOI:** 10.3389/fncir.2014.00100

**Published:** 2014-09-08

**Authors:** James W. Grau, J. Russell Huie, Kuan H. Lee, Kevin C. Hoy, Yung-Jen Huang, Joel D. Turtle, Misty M. Strain, Kyle M. Baumbauer, Rajesh M. Miranda, Michelle A. Hook, Adam R. Ferguson, Sandra M. Garraway

**Affiliations:** ^1^Cellular and Behavioral Neuroscience, Department of Psychology, Texas A&M University, College StationTX, USA; ^2^Department of Neurological Surgery, Brain and Spinal Injury Center, University of California San FranciscoSan Francisco, CA, USA; ^3^Department of Neurosciences, MetroHealth Medical Center and Case Western Reserve UniversityCleveland, OH, USA; ^4^School of Nursing, University of ConnecticutStorrs, CT, USA; ^5^Department of Neuroscience and Experimental Therapeutics, Texas A&M Health Science CenterBryan, TX, USA; ^6^Department of Physiology, Emory University School of MedicineAtlanta, GA, USA

**Keywords:** plasticity, learning, inflammation, spinal cord injury, nociception, BDNF, TNF, opioid

## Abstract

Research has shown that spinal circuits have the capacity to adapt in response to training, nociceptive stimulation and peripheral inflammation. These changes in neural function are mediated by physiological and neurochemical systems analogous to those that support plasticity within the hippocampus (e.g., long-term potentiation and the NMDA receptor). As observed in the hippocampus, engaging spinal circuits can have a lasting impact on plastic potential, enabling or inhibiting the capacity to learn. These effects are related to the concept of metaplasticity. Behavioral paradigms are described that induce metaplastic effects within the spinal cord. Uncontrollable/unpredictable stimulation, and peripheral inflammation, induce a form of maladaptive plasticity that inhibits spinal learning. Conversely, exposure to controllable or predictable stimulation engages a form of adaptive plasticity that counters these maladaptive effects and enables learning. Adaptive plasticity is tied to an up-regulation of brain derived neurotrophic factor (BDNF). Maladaptive plasticity is linked to processes that involve kappa opioids, the metabotropic glutamate (mGlu) receptor, glia, and the cytokine tumor necrosis factor (TNF). Uncontrollable nociceptive stimulation also impairs recovery after a spinal contusion injury and fosters the development of pain (allodynia). These adverse effects are related to an up-regulation of TNF and a down-regulation of BDNF and its receptor (TrkB). In the absence of injury, brain systems quell the sensitization of spinal circuits through descending serotonergic fibers and the serotonin 1A (5HT 1A) receptor. This protective effect is blocked by surgical anesthesia. Disconnected from the brain, intracellular Cl^-^ concentrations increase (due to a down-regulation of the cotransporter KCC2), which causes GABA to have an excitatory effect. It is suggested that BDNF has a restorative effect because it up-regulates KCC2 and re-establishes GABA-mediated inhibition.

## INTRODUCTION

Research has shown that brain systems modulate the operation of spinal circuits. For example, afferent pain (nociceptive) signals can be inhibited, yielding an *anti*-nociception that attenuates both spinally mediated withdrawal and brain-mediated indices of pain (Fields, 2000). This provides a form of top-down processing that allows the organism to dynamically modulate incoming pain signals on the basis of expectation (Grau, 1987; McNally et al., 2011). This type of regulatory effect is characterized as a form of *neuromodulation* because it does not initiate a sensory/motor response, but instead regulates signal amplitude within a spinal circuit to facilitate or inhibit neural transmission. Evidence suggests that how and when these descending systems are engaged is tuned by experience, providing a mechanism whereby brain-mediated learning can influence spinal function (also see: Wolpaw, 2010; Thompson and Wolpaw, 2014).

Here we focus on a different question: can spinal systems learn without input from the brain and is this learning affected by past experience? We will show that how spinal circuits operate depends upon both environmental relations (e.g., the temporal regularity of sensory stimuli) and behavioral control (e.g., a consistent relation between limb position and an environmental stimulus). More importantly, we provide evidence that spinal cord learning affects the propensity to learn in future situations and suggest that this reflects a form of *metaplasticity* (Abraham and Bear, 1996). We will link these metaplastic effects to particular neurochemical systems [e.g., the metabotropic glutamate receptor (mGluR), tumor necrosis factor (TNF), and brain-derived neurotrophic factor (BDNF)]. We will also explore how these processes influence recovery after a spinal contusion injury and how a spinal injury affects their function.

## DRAWING ON PARALLELS TO BRAIN-MEDIATED PROCESSES

### NEURAL PLASTICITY IN THE HIPPOCAMPUS AND SPINAL CORD INVOLVE COMMON MECHANISMS

Our analysis is informed by studies of learning and memory within the brain. Of particular interest are studies of neural plasticity within the hippocampus. Behavioral evidence that this structure is involved in learning and memory (Squire and Wixted, 2011), combined with the physiological findings that this system supports lasting changes in synaptic function [e.g., long-term potentiation (LTP) and long-term depression (LTD); Bear, 2003], have fueled interest in this structure. This work has linked alterations in synaptic function to the NMDA receptor (NMDAR), which acts as a coincidence detector (Collingridge and Bliss, 1987; Dudai, 1989). From this perspective, modifiable (plastic) changes in neural function are identified with synaptic events. While most would acknowledge that neural connections can be altered in a variety of ways, the preponderance of glutamatergic transmission has focused attention on the role of NMDAR-mediated LTP and LTD (Morris, 2013).

Other regions of the central nervous system, including the spinal cord, support NMDAR-mediated plasticity. For example, peripheral injury and inflammation can produce a lasting increase in neural excitability within the spinal cord, a phenomena called *central sensitization* (Woolf, 1983; Willis, 2001; Ji et al., 2003; Latremoliere and Woolf, 2009). Central sensitization lowers the threshold at which stimulation engages a defensive withdrawal response. Indeed, after the system is sensitized, even non-noxious tactile stimulation may elicit a response. Evidence suggests that central sensitization fosters pain transmission to the brain, and for this reason it is thought to contribute to the development of chronic pain. Interestingly, the induction of central sensitization depends upon a form of NMDAR-mediated plasticity that lays down a memory-like alteration that maintains the sensitized state through neurobiological processes analogous to those involved in hippocampal-dependent learning and memory (Dickenson and Sullivan, 1987; Sandkühler, 2000; Ji et al., 2003).

### NEUROMODULATION AND METAPLASTICITY

There is now ample evidence that spinal systems can support some simple forms of learning and memory (reviewed in Grau, 2014). For example, if a rat is spinally transected in the thoracic region and then given a noxious shock to one hindlimb whenever the leg is extended, it learns to maintain the leg in a flexed position (thereby reducing net shock exposure; Grau et al., 1998). Here, learning brings about a modification within a particular stimulus-response (S-R) pathway. What is of greater interest for the present review is that this process of spinal learning can have an effect that impacts the capacity to learn when stimulation is later applied at other sites on the body. For example, experience with controllable stimulation on one leg can foster learning on the contralateral leg whereas exposure to uncontrollable stimulation to either one leg or tail has a lasting inhibitory effect on learning for both legs (Crown et al., 2002a; Joynes et al., 2003).

Correlates to these behavioral observations can be found at the cellular level. For example, electrophysiological stimulation of a neuron can produce a downstream modification (e.g., LTP) that only affects how that particular neural pathway operates. Neural activity can also engage cellular systems that have a remote effect on other neural circuits, providing a form of extrinsic modulation that alters how another neural pathway functions. Our assumption is that environmental stimulation and behavioral training can engage a form of extrinsic modulation that can affect learning (neural plasticity) when stimuli are applied to other regions of the body (and which engage a distinct neural circuit).

How extrinsic processes affect neural function can vary over time. In some cases, a modulatory process may be reflexively elicited in an unconditioned (unlearned) manner and have an acute effect that passively decays over the course of minutes to hours. In other cases, the impact of the modulatory process may continue beyond the events that induced it, to have a long-term effect on how a neural circuit operates. In this case, the initiating event must engage a process that maintains the modulatory process over time, and in this way it involves a kind of memory.

**FIGURE 1:**
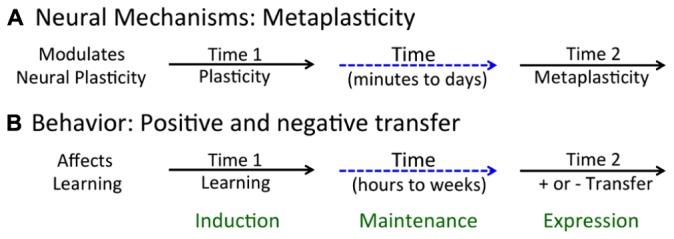
**Acute and long-term effects of neurobiological and behavioral processes. (A)** Metaplasticity arises when an initial event (at Time 1) brings about a lasting change in neural function that affects plastic potential (at Time 2). **(B)** Learning can affect the future capacity to learn, enhancing learning about new events and relations (positive transfer) or inhibiting this process (negative transfer).

A long-term modulatory effect can impact how a neural circuit operates (performs) or its capacity to change (plasticity). Our focus is on the latter, a phenomenon known as *metaplasticity* (Abraham and Bear, 1996). Metaplasticity is a concept that emerged from work with the hippocampal slice preparation and describes a class of phenomena that have a lasting effect on neural plasticity (**Figure 1A**). Here, neural plasticity is typically assessed using electrophysiological processes (e.g., the development of LTP or LTD). What researchers discovered is that a variety of treatments (environmental enrichment, dark rearing, conditioning) can have a lasting effect on the rate at which LTP or LTD develops and may do so without impacting baseline measures of neural excitability (Abraham, 2008). The criteria for metaplasticity include: (1) it extends beyond the treatments used to induce [i.e., it has a lasting effect that spans minutes to days (Abraham, 2008)]; and (2) it impacts the capacity to change (plasticity), not just the responsiveness of the system (performance). To this, we could add another criterion: (3) the phenomenon is reversible (and not due to dysfunction or injury). From this view, an experimental manipulation that permanently alters plastic potential because it kills cells would not be considered an example of metaplasticity.

## EXPERIENCE-DEPENDENT CHANGES IN SPINAL FUNCTION

Our approach begins with a detailed description of the behavioral phenomena and seeks to understand the underlying neurobiological mechanisms. We see this as a complement to physiological approaches that use cellular techniques (e.g., electrophysiology) to detail how components of the system operate. An advantage of the spinal cord preparation is that the link between sensory/motor processes and the underlying neurobiology is (relative to the brain) simpler. For this reason, it may be easier to draw parallels between behavioral effects and neurobiological modifications. In the sections that follow, we show how behavioral manipulations can influence learning potential within the spinal cord and relate these effects to the concept of metaplasticity.

### DEFINING LEARNING

To demonstrate spinal learning requires an operational definition of the process (Grau et al., 1998; Grau, 2010). Learning is implicated when an experience at time 1 has a lasting effect at time 2 (Rescorla, 1988). We formalized this idea by proposing that learning: (1) involves a form of neural plasticity; (2) depends upon the organism’s experiential history; and (3) outlasts (extends beyond) the environmental contingencies used to induce.

While we recognize that non-neural processes (e.g., glia) play an important role, our focus is on how these processes influence neural function (criterion 1). Likewise, while it is recognized that a wide range of events (including development and injury) can engage forms of neural plasticity (Onifer et al., 2011), learning is limited to those engaged by experience (criterion 2). The final requirement (3) is that the process has a lasting effect (which implies a form of memory). From this view, learning reflects the process used to establish a lasting change in neural/behavioral function (memory) and, like most, we assume that the latter generally involves a protein synthesis dependent structural modification (Dudai, 2004).

Whether spinal systems can learn has both theoretical and clinical implications (Grau et al., 2006, 2012; Hook and Grau, 2007; Grau, 2014). Theoretically, it would imply that learning is not the province of particular neural structures within the brain, but instead, is more widely distributed throughout the CNS, including the spinal cord. From this view, the question is not whether a particular system can learn, the question is: how does learning within this system compare to that shown by other structures? Not surprisingly, spinal learning is (relative to the brain) less flexible and more biologically constrained (Grau et al., 2012). Spinal learning is also important because it has implications for physical therapy. Indeed, physical therapy can be seen as a form of directed learning, the aim of which is to establish a lasting change in neural/behavioral function.

Learning phenomena are typically classified based upon the environmental manipulations used to establish the behavioral change (Grau, 2014; Domjan, 2015). For example, Pavlovian conditioning depends upon the relation between two stimulus events whereas instrumental learning is tied to the relation between a behavioral response (R) and an environmental event [the outcome (O); aka reinforcer]. Recognizing that physical therapy typically involves a kind of instrumental training, we asked whether neurons within the lumbosacral spinal cord are sensitive to response–outcome (R–O) relations (Grau et al., 1998, 2006).

### SPINALLY MEDIATED INSTRUMENTAL LEARNING

The first clear evidence that instrumental learning can produce a lasting modification in spinal function was provided by Wolpaw and Carp (1990) and Wolpaw (2010). The response involved a modification of the spinal stretch reflex [the Hoffman (H) reflex] and change in reflex magnitude was reinforced with food. For example, in some subjects the H-reflex was repeatedly elicited and they were reinforced for exhibiting an increase in response strength. This training brought about an increase in the H-reflex. Remarkably, after extended training, this response modification survived a spinal transection. This work demonstrates that instrumental learning can modify spinal function. Here, brain mechanisms mediate the abstraction of the instrumental relation [between H reflex amplitude (the R) and the food reinforcer (the O)]. With extended training, this R–O relation induces (through descending fibers) a lasting change in how a spinal circuit operates. In this case, learning is mediated by the brain and the consequence of this process (the memory) is stored within the spinal cord.

**FIGURE 2:**
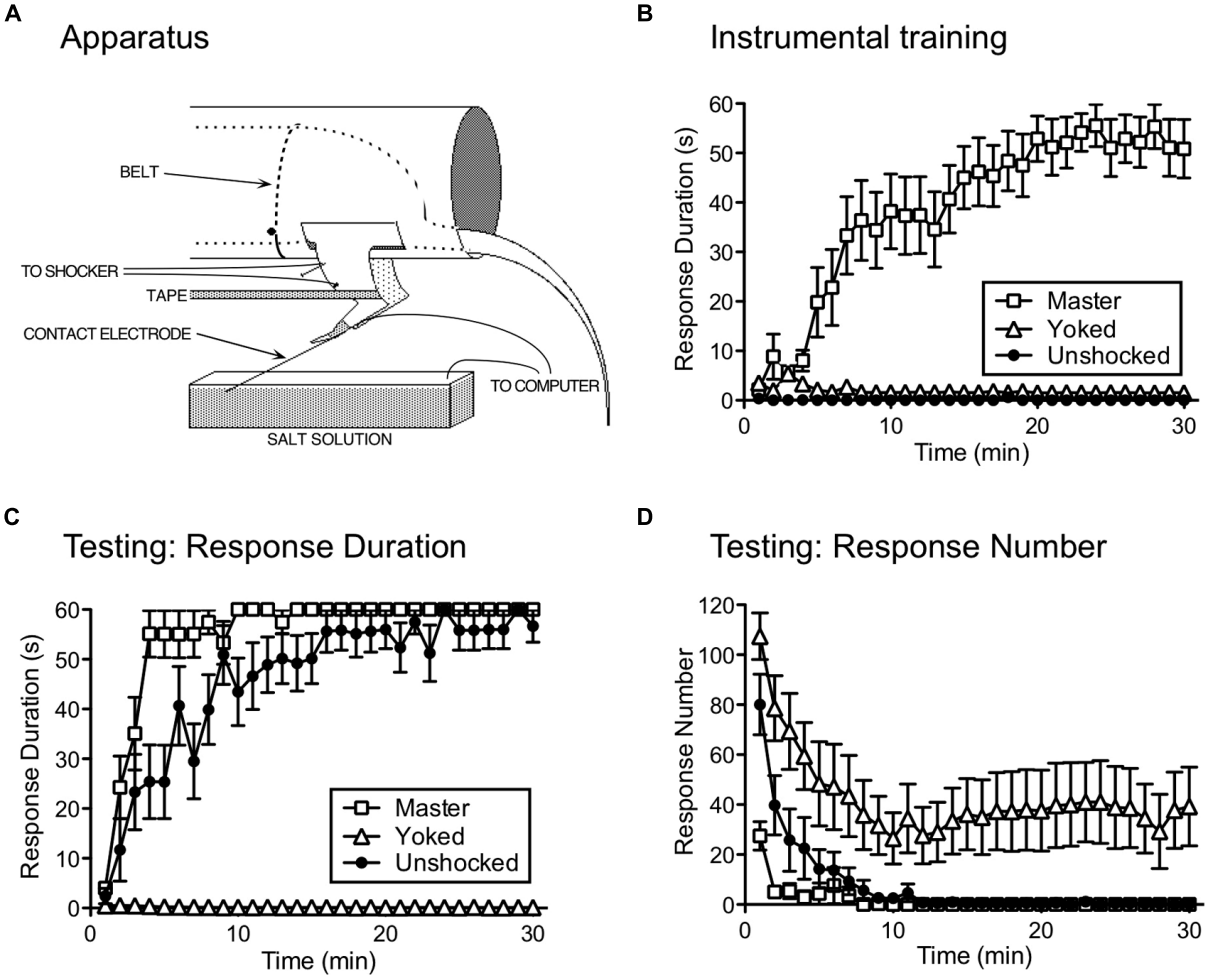
**Instrumental learning in spinally transected rats. (A)** The apparatus used to study instrumental learning. A spinally transected rat lies in an opaque tube with its hindquarters gently secured with a belt. An insulated contact electrode is taped to the rat’s paw and the exposed tip is submerged in a salt solution. Electrical stimulation is applied to the tibialis anterior muscle through a pair of electrodes and tape is used to stabilize the leg. When shock is applied, a flexion response is elicited that raises the contact electrode, breaking a circuit that is monitored by a computer. A response–outcome (R–O) relation is instituted by applying shock whenever the contact electrode touches the underlying salt solution. The task can be made more difficult by increasing the initial depth of the contact electrode (from 4 to 8 mm). **(B)** A system capable of learning the R–O relation should exhibit an increase in flexion (response) duration that minimizes solution contact (and net shock exposure). Response duration (y-axis) is calculated in 1-min time bins using the following formula: Response duration = [60-time (s) in solution]/(flexion number + 1). Over the course of 30 min of testing (x-axis), spinally transected rats that received shock whenever the leg was extended (Master) exhibited a progressive increase in response duration. Other rats are experimentally coupled (Yoked) to the master subjects and receive shock at the same time, but independent of leg position (uncontrollable stimulation). Yoked rats do not exhibit an increase in response duration. The error bars indicate the standard error of the mean. **(C)** Master, Yoked, and previously unshocked rats are then tested under common conditions with controllable shock. Master rats learn more rapidly (positive transfer) than the previously untreated (Unshocked) controls. Rats that had previously received shock independent of leg position (Yoked) fail to learn. Similar results are observed independent of whether subjects are tested on the previously trained (ipsilateral) leg or the contralateral leg. **(D)** As Master rats learn to maintain their leg in a flexed position, response number declines. In Yoked rats, shock elicits a high response rate, but does not produce an increase in flexion duration. Adapted from Grau et al. (1998).

Our studies pushed spinal systems further, to explore whether neurons within the lumbosacral cord can learn (i.e., abstract R–O relations) when isolated from the brain. Rats underwent a thoracic (T2) transection and were trained the following day while loosely restrained (**Figure 2A**). Leg position is monitored by means of a contact electrode that is taped to the base of the hindpaw. When the leg is extended, the tip of the contact electrode touches the underlying salt solution and completes a computer-monitored circuit. A R–O relation is then established by applying shock to the tibialis anterior muscle whenever the leg is extended. Over the course of 30 min of training, subjects exhibit a progressive increase in flexion duration that minimizes net shock exposure (**Figure 2B**; Grau et al., 1998). This learning depends upon glutamatergic systems within the spinal cord, and is blocked when an AMPAR (CNQX) or NMDAR (APV; MK-801) antagonist is injected into the spinal cord [an intrathecal (i.t.) injection] prior to training (Joynes et al., 2004; Ferguson et al., 2006; Hoy et al., 2013).

To show that the R–O relation matters, other subjects received shock independent of leg position. This was accomplished by coupling (yoking) the experimental treatments across subjects, so that a yoked rat received shock every time its master partner was shocked. Notice that, for the yoked rat, there is no relation between shock exposure and leg position – the shock is uncontrollable. Subjects in the yoked group do not exhibit an increase in flexion duration (**Figure 2B**), which provides one indication that the R–O relation matters.

To demonstrate learning, we must show that the experience has a lasting effect, that impacts performance when subjects are tested under common conditions. We accomplished this by testing rats that had previously received controllable shock (Master), uncontrollable shock (Yoked), or nothing (Unshocked) with response contingent legshock. We were concerned that yoked rats might do poorly during testing simply because they were less responsive to shock or the contact electrode was submerged at a greater depth. To discount these factors, we adjusted shock intensity across subjects so that it elicited an equally strong flexion response and equated contact electrode depth (to 4 mm). We verified the success of these procedures by measuring by measuring the duration of the first shock-elicited flexion response. As expected, there were no differences in performance at the start of testing. Nonetheless, subjects that had previously experienced controllable stimulation learned faster than previously unshocked controls (**Figure 2C**; Grau et al., 1998). This savings effect (positive transfer; **Figure 1B**) provides one indication that training with response-contingent stimulation has a lasting effect. What was more surprising is that rats that had previously received uncontrollable stimulation (Yoked) failed to learn when later tested with controllable shock (negative transfer). Moreover, they failed to learn even though they exhibited a high rate of responding and repeatedly experienced the R–O relation (**Figure 2D**).

### UNCONTROLLABLE STIMULATION AND INFLAMMATION INDUCE A LASTING LEARNING IMPAIRMENT

Does the learning impairment observed after uncontrollable stimulation to one hind limb reflect a local (limb-specific) effect or a general inhibition of learning? We addressed this issue by testing yoked subjects on the same (ipsilateral) or opposite (contralateral) leg. The learning impairment was just as robust when subjects were tested on the contralateral leg (Joynes et al., 2003). Next, we developed a computer program that emulated the variable shock schedule produced by a typical master rat. This program applies 80 ms shocks with a variable inter-stimulus interval (ISI) between 0.2 and 3.8 s (mean ISI = 2 s). We found that just 6 min of variable intermittent shock (VIS) to the leg or tail induced a learning impairment and that this effect lasts up to 48 h (Crown et al., 2002b). Thus, exposure to uncontrollable stimulation induces a lasting effect that generally inhibits instrumental learning. We have suggested that this learning deficit reflects a form of metaplasticity (Ferguson et al., 2008, 2012a).

We reasoned that uncontrollable stimulation could inhibit learning because it induces a form of antinociception that attenuates the effectiveness of the shock reinforcer. However, we found no evidence that VIS inhibits reactivity to noxious stimulation (Crown et al., 2002b). If fact, a test of mechanical reactivity (von Frey stimuli applied to the plantar surface of the hind paws) showed that VIS treated subjects were more responsive (Ferguson et al., 2006). Enhanced mechanical reactivity (EMR) is of interest because it is observed after a variety of treatments known to sensitize nociceptive systems within the spinal cord (central sensitization).

As noted above, central sensitization involves neurochemical mechanisms implicated in hippocampal-dependent learning and memory and its induction depends upon glutamate transmission and the NMDAR (Ji et al., 2003). We hypothesized that this state could interfere with instrumental learning by saturating NMDAR-dependent plasticity (Ferguson et al., 2006). Alternatively, the induction of central sensitization could engage a secondary process that inhibits NMDAR-mediated learning, effectively “locking” the system in its current state. In either case, blocking the NMDAR should interfere with the induction of the learning impairment. Supporting this, we found that rats given MK-801 prior to VIS showed no learning impairment when tested with controllable stimulation 24 h later (Ferguson et al., 2006). Pretreatment with the AMPAR antagonist CNQX had a similar effect (Hoy et al., 2013).

The proposed link to central sensitization suggests that treatments that induce this state should impair instrumental learning. To test this, we applied the irritant capsaicin to one hind paw, which induces peripheral inflammation and central sensitization (Willis, 2001). Capsaicin also induced a learning impairment and this effect, like the VIS-induced deficit, was evident 24 h later when subjects were tested on the contralateral leg (Hook et al., 2008; for evidence other inflammatory agents inhibit learning see Ferguson et al., 2006, 2012b; Huie et al., 2012a).

### CONTROLLABLE STIMULATION FOSTERS LEARNING AND HAS A LASTING PROTECTIVE EFFECT

Whereas uncontrollable stimulation and peripheral inflammation disable learning, controllable stimulation enables instrumental learning (Crown et al., 2002a). Evidence for this comes from studies using a higher response criterion, achieved by increasing contact electrode depth (from 4 to 8 mm). Under these conditions, previously untrained rats fail to learn whereas those that had received controllable stimulation can learn and this is true independent of whether they are tested on the same or opposite leg.

Controllable stimulation also exerts a protective effect that counters the consequences of uncontrollable shock. If controllable stimulation is given prior to VIS (to the same leg or the tail), it blocks the induction of the learning impairment (Crown and Grau, 2001). Conversely, after the learning impairment is induced, training with controllable shock [in conjunction with a drug treatment (naltrexone) that temporarily reverses the impairment (see below)] restores the capacity to learn (when subjects are subsequently tested in a drug-free state). Exposure to controllable shock also prevents, and reverses, the learning impairment and EMR induced by peripheral capsaicin (Hook et al., 2008).

The fact controllable stimulation enables learning when subjects are tested on the opposite leg, and prevents the learning impairment when VIS is applied to the tail, implies that controllable stimulation generally modulates the capacity to learn. Further, we have shown that instrumental training has a lasting effect that can block the induction of the learning deficit when VIS is given 24 h later. Taken together, these findings suggest that exposure to controllable stimulation also induces a metaplastic effect, one that promotes instrumental learning.

We, of course, are not the first to show that behavioral control can profoundly affect how an aversive stimulus is processed. Indeed, the overall pattern of results is remarkably similar to what is observed in intact subjects in studies of learned helplessness (Maier and Seligman, 1976). These observations suggest that that the underlying principles have considerable generality and may apply to any neural system capable of encoding R–O relations. At the same time, it is also recognized that higher neural systems allow for a much wider range of behavioral effects (Maier and Watkins, 2005) and that spinal learning is more biologically constrained (Grau et al., 2012).

### TEMPORAL REGULARITY (PREDICTABILITY) HAS AN EFFECT ANALOGOUS TO BEHAVIORAL CONTROL

We recently discovered that uncontrollable intermittent shock does not always induce a learning impairment. If stimulation is given at a regular (predictable) interval, an extended exposure to intermittent shock has no adverse effect (Baumbauer et al., 2008). Interestingly, the emergence of this effect requires extended training (720–900 shocks); if subjects receive less training (180 shocks), intermittent shock induces a learning impairment independent of whether it occurs in a variable or regular (fixed-spaced) manner. The fact extended training is required has led us to suggest that abstracting stimulus regularity involves a form of learning (Baumbauer et al., 2009).

At a behavioral level, we have shown that an initial bout of fixed spaced shock (360) lays down a kind of temporal memory that lasts at least 24 h and transforms how subjects respond to a subsequent bout of 360 shocks (processing the latter as fixed spaced; Lee et al., 2013). At a physiological level, we have shown that learning about temporal regularity depends upon a form of NMDAR-mediated plasticity and protein synthesis (Baumbauer et al., 2009). Further, training with fixed spaced stimulation has a restorative effect analogous to that produced by experience with controllable stimulation. For example, fixed spaced stimulation can both prevent, and reverse, the learning impairment induced by VIS (Baumbauer et al., 2009). An extended exposure to fixed spaced shock also blocks, and reverses, the learning impairment and EMR induced by peripheral inflammation (Baumbauer and Grau, 2011; Baumbauer et al., 2012). And like the other effects described above, fixed spaced stimulation has a general effect that blocks the induction of the learning impairment independent of whether subjects are challenged by stimulation at the same, or a remote, dermatome (Baumbauer et al., 2009).

### SPINAL LEARNING: SUMMARY AND IMPLICATIONS

**FIGURE 3:**
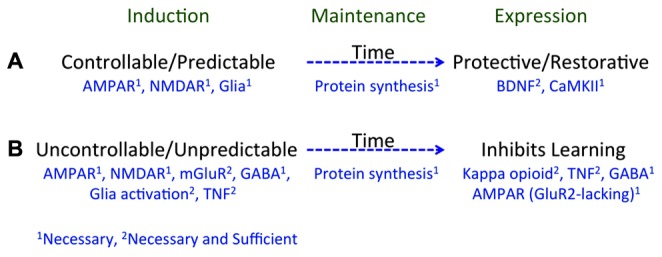
**A summary of how training experience affects learning potential. (A)** Learning that an environmental event is controllable or predictable depends upon a form of NMDAR-mediated plasticity and is disrupted if glia function is inhibited. Processes initiated during learning (induction) engage a protein synthesis dependent mechanism that maintains the effect over time (memory). Prior experience with controllable/predictable stimulation has a long-term protective/restorative effect that enables instrumental learning and blocks both the learning impairment and EMR induced by VIS. These effects depend upon BDNF and CaMKII. **(B)** Exposure to uncontrollable/unpredictable shock initiates a process that depends upon the NMDAR, group 1 mGluR, GABA, glia activation, and TNF. These processes induce a protein synthesis dependent mechanism that maintains the effect over time. Prior experience with uncontrollable/unpredictable stimulation inhibits instrumental learning through a process that involves a kappa opioid, TNF, GABA, and the trafficking of GluR2-lacking AMPA receptors. Superscripts indicate whether a neurobiological mechanism is necessary (1) or necessary and sufficient (2).

Taken together, we have discovered that environmental events can engage two alternative processes that have a diffuse effect on spinal cord plasticity (**Figure 3**; for reviews see: Grau et al., 2006, 2012; Ferguson et al., 2012a). Exposure to VIS, that is both uncontrollable and unpredictable, inhibits instrumental learning and produces EMR (Grau et al., 1998; Baumbauer et al., 2008), and peripheral inflammation has the same effect (Hook et al., 2008). An equivalent exposure to intermittent stimulation given in a controllable manner has no adverse effect and engages a process that enables learning and counters the adverse effects of both VIS and inflammation (Crown and Grau, 2001; Crown et al., 2002a). Likewise, an extended exposure to fixed spaced shock engages a protective mechanism that counters the adverse effects of VIS (Baumbauer et al., 2009, 2012; Baumbauer and Grau, 2011). These effects are lasting (24 h or longer), involve a form of NMDAR-mediated plasticity, and require protein synthesis (Joynes et al., 2004; Patton et al., 2004; Baumbauer et al., 2006, 2009; Ferguson et al., 2006). Moreover, in all cases the phenomena have a general effect that impacts how stimuli applied at other dermatomes are processed.

We have seen that learning can both alter a particular response and impact the capacity to learn when faced with new environmental challenges (Grau et al., 1998; Crown et al., 2002a). Our focus here is on the latter phenomena – on how learning can foster (positive transfer), or inhibit (negative transfer), the capacity for future learning (**Figure 1B**). In assuming that learning involves a form of neural plasticity, the question we ask focuses on the plasticity of plasticity: How does a training experience impact the future capacity to learn? We suggest that this reflects a form of metaplasticity.

In the sections that follow, we outline what we have discovered about the neurobiological mechanisms that mediate these metaplastic effects. While we will reference electrophysiological observations, our discussion will lean towards an analysis of behavioral indices of spinal function. We will also remain agnostic regarding the relation of our effects to the phenomena of LTP and LTD. We take this position because we have yet to elucidate the relative role of these phenomena and because we assume that neural plasticity may be mediated by a host of mechanisms.

## THE BIOLOGY OF SPINALLY MEDIATED METAPLASTICITY

### LINKING METAPLASTICITY TO MECHANISM

Our central concern is with processes that have a lasting effect and, in this way, involve a form of memory. It is assumed here that acute changes in neural function are mediated by pre-existing components and that long-term modifications depend upon protein synthesis (Dudai, 2004; Abraham and Williams, 2008). This holds for our examples of spinally mediated metaplasticity. Supporting this, administration of a protein synthesis inhibitor soon after exposure to VIS blocks the induction of the learning impairment (Patton et al., 2004; Baumbauer et al., 2006). Likewise, administration of a protein synthesis inhibitor after a fixed spaced shock blocks its long-term protective effect (Baumbauer et al., 2009).

Because these metaplastic effects involve a form of memory, we can address the process from a number of perspectives (**Figure 1**). Specifically, we can ask: (1) What processes underlie the *induction* of the phenomenon; (2) What mediates the *maintenance* of the alteration (the memory) over time; and (3) What mediates the *expression* of these phenomena (i.e., how do they affect the capacity to learn)? We address question 3 by blocking a particular process (necessity) and then showing that administration of an agent that should engage the process has a similar effect on learning (sufficiency). The interpretation of sufficiency must, though, be treated with some caution because engaging other (unrelated) processes could yield a similar outcome. For the second issue, the question typically concerns the identification of the neurobiological system that preserves the effect over time. To study the induction of the process (question 1), we can again assay the effect of blocking a particular process, seeking evidence that it plays an essential (necessary) role. For evidence of sufficiency, we can test whether artificially engaging the system effectively substitutes for our experimental treatment. Again, some caution is needed because a similar outcome may be produced in a variety of ways. Further, the induction of most phenomena is tied to multiple processes. In this case, to discover a substitute for a behavioral training regime, we need to know all of the essential components and how they are sequenced over time.

The link between the learning impairment and central sensitization has provided a rich source of concepts regarding the neurobiological mechanisms that may be involved, implicating opioid peptides, glutamatergic transmission (AMPAR, NMDAR, and mGluR), non-neuronal cells and TNF. Identifying the factors that promote adaptive plasticity has proven more difficult. We have, however, discovered that BDNF, and downstream signal pathways (e.g., CaMKII), play an important role.

### ROLE OF THE NMDAR AND mGluR IN THE INDUCTION OF THE LEARNING IMPAIRMENT

We noted above that both instrumental learning and the metaplastic effects of training depend upon the NMDAR. Supporting this, pretreatment with a NMDAR antagonist disrupts instrumental learning, the long-term protective effect of fixed spaced stimulation, and the induction of the learning impairment (Joynes et al., 2004; Ferguson et al., 2006; Baumbauer et al., 2009). We have also examined whether pretreatment with NMDA has a long-term effect on learning. While a high dose of NMDA (6 mM, 15 μL i.t.) induced a lasting learning impairment (Ferguson et al., 2012b), moderate doses (e.g., 0.06–0.6 mM) that are within the range that foster locomotor behavior have no long-term effect (Strain et al., 2013). Because NMDA was only effective at a high concentration, it possible that it impaired plasticity because it induced a non-reversible state. Before we conclude that NMDA is sufficient to induce a VIS-like learning impairment, we need to address this issue. For now, we can conclude only that the NMDAR plays a necessary role. This is true for a wide range of spinal learning phenomena, including sensitization, Pavlovian conditioning, instrumental learning, and the metaplastic effects of training discussed here (Durkovic and Prokowich, 1998; Willis, 2001; Ji et al., 2003; Joynes et al., 2004; Ferguson et al., 2006).

**FIGURE 4:**
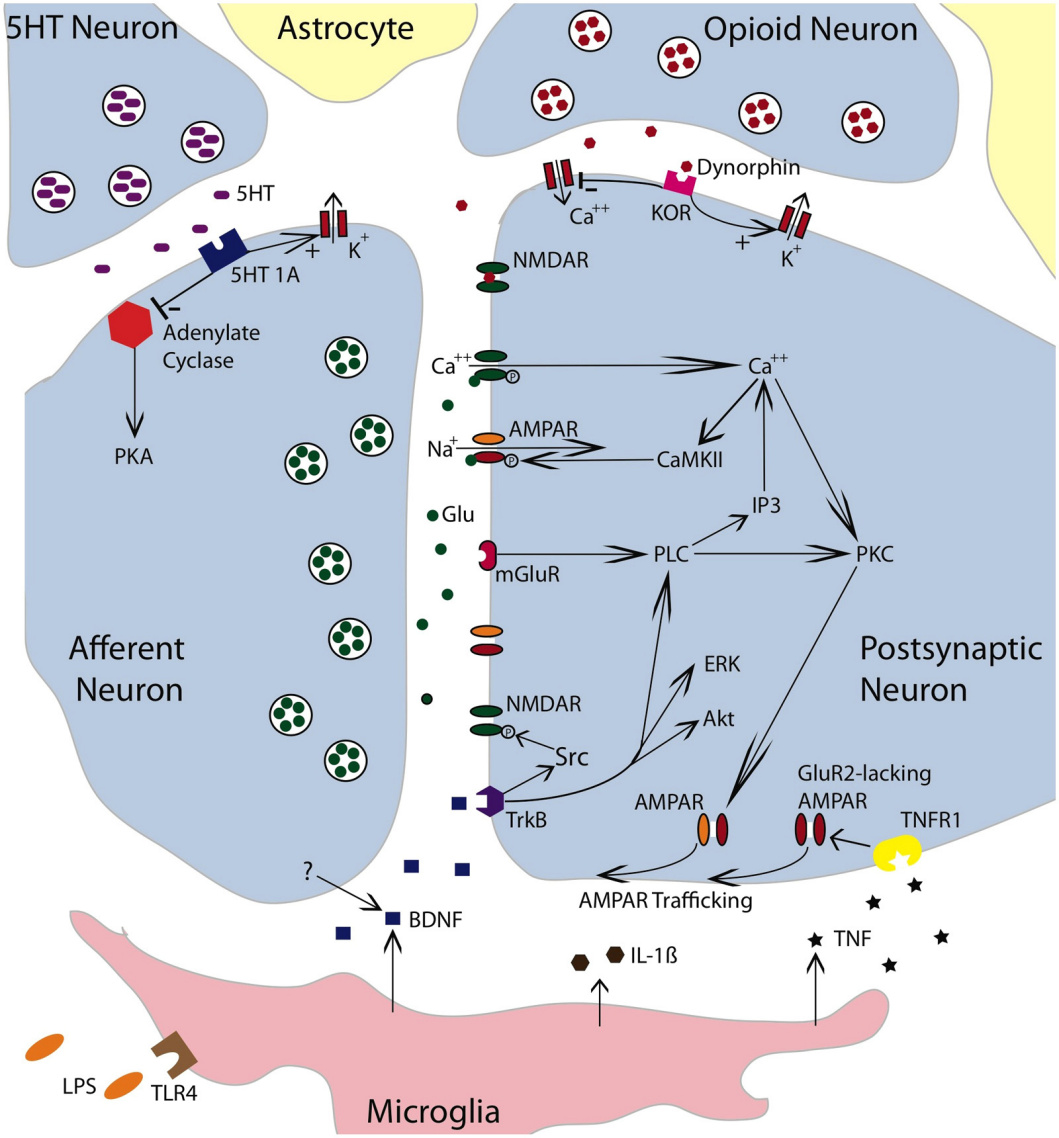
**Neurochemical mechanisms involved in spinally mediated learning and metaplasticity.** The figure depicts a tripartite synapse involving an afferent neuron, a postsynaptic neuron, and the surrounding astrocytes. The afferent neuron is glutamatergic. Released glutamate (Glu) can engage the NMDAR, AMPAR, or mGluR receptors on the postsynaptic neuron. Activating the NMDAR in conjunction with a strong depolarization allows Ca^++^ to enter the postsynaptic cell, which engages intracellular signals such as Ca^++^/calmodulin-dependent protein kinase (CaMKII) and protein kinase C (PKC). CaMKII activates the AMPAR and thereby promotes the entry of Na^+^. Engaging the mGluR activates phospholipase C (PLC), which engages inositol triphosphate (IP3) and PKC. IP3 initiates the release of intracellular Ca^++^. PKC promotes the trafficking of AMPARs to the active region of the synaptic membrane. To illustrate the relevant pathways, cells that exert a modulatory effect are also indicated. These include a descending serotonergic (5HT) neuron, an kappa opioid neuron, and a microglia. Both the 5HT neuron and opioid neuron would exert an inhibitory effect that could act on either the presynaptic or postsynaptic neuron. Dynorphin released from the opioid neuron would engage the kappa opioid receptor (KOR), which would inhibit neural excitation by facilitating the flow of K^+^ out of the cell and inhibiting the inward flow of Ca^++^. Dynorphin can also bind to the NMDAR in its closed state and thereby inhibit NMDAR function. Engaging the 5HT 1A receptor would inhibit adynylate cyclase. This reduces the conversion of adenosine triphosphate (ATP) to cyclic adenosine monophosphate (cAMP) and down-regulates cAMP-dependent processes [e.g., protein kinase A (PKA)]. Promoting the outward flow of K^+^ would reduce neural excitability. Lipopolysaccharide (LPS) can engage the toll-like receptor 4 (TLR4) and activate microglia. Microglia have been shown to release TNF, IL-1β, and BDNF. BDNF may also be released from neurons. BDNF can foster NMDAR function through Src kinase. It also promote plasticity by engaging extracellular signal regulated kinase (ERK), serine-threonine-specific protein kinase (Akt) and PLC. By engaging the TNFR1, TNF fosters the trafficking of GluR2-lacking AMPARs to the synaptic membrane. The simplified drawing omits details (e.g., glutamatergic channels on microglia) that could contribute to spinally mediated metaplasticity. Adapted from Ji et al. (2003, 2013), Grau et al. (2006), and Cunha et al. (2010).

**FIGURE 5:**
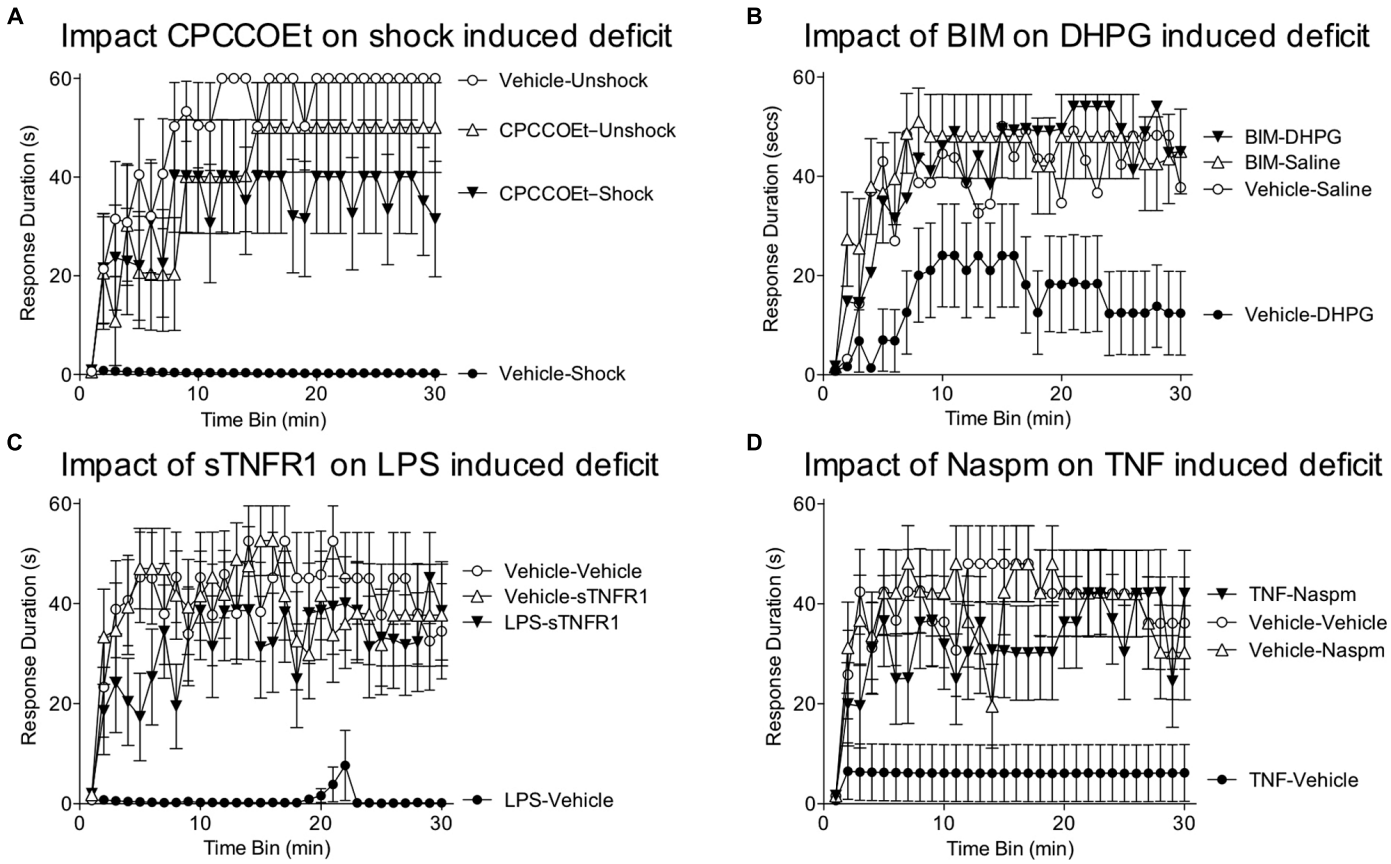
**Role of group 1 mGluR, glia, and TNF in the learning deficit. (A)** Spinally transected rats received the group 1 mGluR1 antagonist CPCCOEt (i.t.; 100 nmol), or its vehicle, prior to VIS (Shock). Instrumental learning was tested 24 h later. Prior exposure to shock inhibited learning (Vehicle-Shock). Pretreatment with CPCCOEt blocked the induction of this learning impairment (CPCCOEt-Shock). **(B)** Rats were pretreated with the PKC inhibitor BIM (i.t.; 0.023 nmol), or its vehicle, and then received an injection of the group 1 mGluR agonist DHPG (100 nmol) or saline. Administration of DHPG alone (Vehicle-DHPG) impaired learning and this effect was blocked by pretreatment with BIM (BIM-DHPG). **(C)** Subjects received an intrathecal injection of LPS (100 μg), or its vehicle. The next day, a TNF inhibitor (sTNFR1; 700 ng) or vehicle was given intrathecal and subjects were tested in the instrumental learning paradigm. Prior treatment with LPS impaired learning (LPS-Vehicle). Administration of sTNFR1 prior to testing eliminated the learning deficit (LPS-sTNFR1). **(D)** Rats received an intrathcal injection of TNF (6000 pg) or its vehicle. The next day, they were given the GluR2 antagonist Naspm (i.e., 10 mM) or vehicle and tested in the instrumental learning paradigm. Rats that had previously received just TNF (TNF-Vehicle) failed to learn. This learning impairment was blocked by Naspm (TNF-Naspm). Adapted from Ferguson et al. (2008) and Huie et al. (2012a).

Evidence suggests that glutamate within the hippocampus can induce a metaplastic effect by engaging the mGluR (Cohen et al., 1999). Of particular interest, activation of group I mGluRs has been shown to facilitate both the induction and persistence of LTP within area CA1 (Abraham, 2008). This effect appears to be mediated by a number of mechanisms, including the trafficking of AMPARs to the synaptic membrane and the amplification of NMDAR-mediated currents (**Figure 4**; O’Connor et al., 1994; MacDonald et al., 2007). It has also been suggested that activating mGluRs can engage a “molecular switch” that enhances the persistence of LTP through a process that depends on group 1 mGluRs and PKC (Bortolotto et al., 1994). Within the spinal cord, group 1 mGluR antagonists have been shown to attenuate inflammation-induced EMR (Stanfa and Dickenson, 1998; Neugebauer et al., 1999; Karim et al., 2001; Zhang et al., 2002) and group 1 mGluR activity has been implicated in the development of neuropathic pain and tissue loss after spinal cord injury (SCI; Mukhin et al., 1996; Agrawal et al., 1998; Mills and Hulsebosch, 2002; Mills et al., 2002). Given these observations, we hypothesized that group 1 mGluR activity contributes to the induction of the learning impairment (Ferguson et al., 2008). Recognizing that two group 1 mGluR subtypes (mGluR1 and mGluR5) have been shown to impact hippocampal plasticity, we evaluated the effects of both CPCCOEt (a mGluR1 antagonist) and MPEP (a mGluR5 antagonist). After intrathecal application of the drug, subjects received 6 min of VIS and instrumental learning was tested 24 h later. We found that both drugs blocked the induction of the learning impairment in a dose-dependent manner (**Figure 5A**). We also examined whether either drug disrupted learning. Neither did and, if anything, CPCCOEt facilitated learning. These findings suggest that activation of group 1 mGluRs is necessary to the induction of the learning impairment. Finally, we asked whether mGluR activation in the absence of VIS is sufficient to induce a learning impairment. Subjects received the group 1 mGluR agonist DHPG and were tested 24 h later. We found that pretreatment with DHPG induced a lasting learning impairment.

Other work suggests that group 1 mGluRs can impact synaptic function through a PKC-mediated signal cascade (Aniksztejn et al., 1992; Skeberdis et al., 2001). We assessed PKC activation and observed enhanced activity one hour after treatment (Ferguson et al., 2008). Further, pretreatment with two structurally distinct PKC inhibitors (BIM; chelerythrine) blocked the learning impairment induced by VIS and DHPG (**Figure 5B**). Importantly, BIM had no effect on instrumental learning. Taken together, the findings suggest that the long-term metaplastic effect of VIS on spinal plasticity involves mGluR activation and PKC.

### KAPPA OPIOIDS MEDIATE THE EXPRESSION OF THE LEARNING IMPAIRMENT

Prior work has shown that intact subjects exposed to uncontrollable stimulation exhibit a learning/performance deficit in instrumental learning tasks, a phenomenon known as learned helplessness (Maier and Seligman, 1976). Evidence suggests that the performance deficit is mediated, in part, by the release of an endogenous opioid (Maier, 1986). Supporting this, administration of an opioid antagonist (naltrexone) prior to testing attenuates the behavioral impairment observed in a shuttle avoidance task (Blustein et al., 1992). Likewise, we found that intrathecal administration of naltrexone attenuates the learning impairment observed in spinally transected rats that had received VIS (Joynes and Grau, 2004). We further showed that naltrexone is effective when given prior to testing, but has no effect when given the day before uncontrollable stimulation. This implies that a ligand that acts on a naltrexone-sensitive receptor plays an essential role in the expression of the learning impairment, but is not involved in its induction.

Because naltrexone is a relatively non-selective opioid antagonist, we also assessed the impact of drugs that bind to the mu (CTOP), delta (naltrindole), and kappa (nor-BNI) opioid receptors (Washburn et al., 2008). Using intrathecal administration of equal molar concentrations we showed that the expression of the learning impairment is blocked by a kappa receptor antagonist, but not a mu or delta antagonist. Conversely, intrathecal administration of a kappa-2 agonist (GR89696) impairs learning, whereas a mu (DAMGO) or a delta (DPDPE) agonist has no effect. Interestingly, a kappa-1 agonist (U69593) also had no effect on learning. Finally, we tested whether pretreatment with a kappa-2 agonist could substitute for VIS and induce a long-term learning impairment. It did not.

These observations suggest that the expression of the learning impairment is mediated by a ligand that acts at the kappa opioid receptor (**Figure 4**), possibly due to a kappa-2 mediated inhibition of NMDAR-mediated synaptic plasticity (Wagner et al., 1993; Caudle et al., 1994, 1997; Ho et al., 1997). Alternatively, kappa-2 opioid activity may “lock” the system in its current state, reducing plastic potential (Washburn et al., 2008). We noted above that the induction of the learning impairment is blocked by pretreatment with an NMDAR antagonist. If kappa opioids inhibit NMDAR-mediated plasticity, administration of a kappa agonist prior to VIS should interfere with the induction of the learning impairment. Washburn et al. (2008) found that GR89696 had this effect.

We suggested above that exposure to uncontrollable stimulation, or peripheral inflammation, may inhibit instrumental learning because these manipulations diffusely saturate NMDAR-mediated plasticity (Ferguson et al., 2006). If that alone was the cause of the learning impairment, there would be little reason to expect an opioid antagonist to block the expression of the learning impairment. Opioid reversibility implies that NMDAR-mediated plasticity remains functional, because as soon as the opioid brake is removed, learning can proceed. At a minimum, the observation requires a more sophisticated view of the factors that limit neural plasticity, that goes beyond the trafficking of AMPAR’s, because it seems unlikely that an opioid antagonist could undo this effect within minutes of administration. These observations are also important for our claim that the learning impairment reflects a form of metaplasticity because the best examples of this phenomena involves cases wherein the underlying plasticity remains functional (Abraham, 2008).

### GLIA AND TNF CONTRIBUTE TO SPINAL LEARNING IMPAIRMENTS

Throughout the nervous system, glia regulate synaptic efficacy, leading some to suggest the concept of a tripartite synapse (**Figure 4**; Araque et al., 1999; Haydon, 2001). In the spinal cord, glial activation plays an essential role in the development of inflammation-induced EMR (Meller et al., 1994; Watkins et al., 1997). Glia can be activated by administration of lipopolysaccharide (LPS) and, when applied intrathecally, this induces EMR (Reeve et al., 2000).

To examine whether glial activation is essential to spinal learning, we tested the effect of fluorocitrate. Fluorocitrate inhibits aconitase, an essential component of the tricarboxylic acid cycle within glia, and thereby disrupts energy-dependent transmitter up-take and release (Paulsen et al., 1987). If glia are essential to spinal plasticity, intrathecal fluorocitrate should inhibit instrumental learning. We found that fluorocitrate does so in a dose-dependent manner (Vichaya et al., 2009). Next, we administered fluorocitrate prior to VIS and tested subjects 24 h later. We found that drug treatment blocked the induction of the learning impairment. Further, intrathecal application of LPS substituted for VIS and interfered with instrumental learning when subjects were tested 24 h later. This long-term effect of LPS was blocked by pretreatment with fluorocitrate. These findings provide further evidence that glia regulate spinal plasticity. More importantly, the results show that glia activation contributes to the long-term consequences of shock treatment; that glial activation is necessary, and sufficient, to the induction of a lasting inhibition of neural plasticity.

Glia can regulate synaptic plasticity through the release of cytokines, such as TNF and interleukin-1. TNF is of particular interest because it is known to modulate synaptic plasticity in hippocampal sections (Stellwagen and Malenka, 2006) and plays an essential role in the development of central sensitization (Czeschik et al., 2008; Park et al., 2011). TNF could interfere with learning by increasing the trafficking of AMPARs to the post-synaptic membrane (Beattie et al., 2002), an effect that has been linked to an up-regulation of Ca^++^ permeable GluR2-lacking AMPARs that increase postsynaptic excitability. If driven too far, this could potentially lead to excitotoxicity enhanced cell death after spinal injury (Ferguson et al., 2008). To explore whether TNF contributes to the learning impairment, we administered the soluble TNF receptor (sTNFR1), which inhibits TNF function by binding free TNF (Huie et al., 2012a). sTNFR1 was given intrathecal prior to VIS (induction phase) or 24 h later prior to testing (expression). sTNFR1 blocked both the induction and the expression of the learning impairment. Next, we asked whether administration of TNF would substitute for VIS treatment. We found that intrathecal TNF impaired learning when subjects were tested 24 h later. Mirroring the long-term effect of VIS treatment, the expression of the TNF-induced learning deficit was blocked by sTNFR1. sTNFR1 also blocked the expression of the learning deficit induced by LPS (**Figure 5C**). Likewise, inhibiting glial activation (with fluorocitrate) prior to TNF treatment blocked the induction of the learning impairment. These observations suggest that TNF has a long-term effect by activating glia and that this in turn enhances subsequent TNF release (Kuno et al., 2005). Cellular assays verified that TNF protein expression was increased 24 h after treatment with VIS (Huie et al., 2012a).

TNF could over-drive neural excitability by increasing the proportion of GluR2-lacking AMPARs. If this is how TNF interferes with learning, administering an antagonist (Naspm) that blocks these AMPARs should reinstate the capacity to learn. To test this, we induced a learning impairment with VIS or intrathecal TNF. The next day, subjects were given Naspm, or its vehicle, and tested in our instrumental learning paradigm. As expected, both TNF and VIS impaired learning. In both cases, treatment with Naspm reinstated the capacity to learn (**Figure 5D**). On-going studies are examining whether VIS reduces the proportion of synaptic AMPARs that contain the GluA2 subunit (Stuck et al., 2012).

In summary, our finding suggests that spinal plasticity depends on glia. Further, VIS appears to induce a lasting learning impairment by engaging glia and up-regulating the release of TNF. We suggest that TNF impairs learning, perhaps by increasing the proportion of Ca^++^ permeable (GluR2-lacking) AMPARs. This could induce a state of over-excitation that interferes with learning, contributes to EMR, and promotes cell death after injury.

### BDNF MEDIATES THE BENEFICIAL EFFECT OF TRAINING

We now understand a great deal about how VIS has a lasting effect on spinal plasticity, with evidence implicating the mGluR, glia, and TNF (Ferguson et al., 2008; Vichaya et al., 2009; Huie et al., 2012b). As discussed below, these observations have clinical implications. But of potentially greater long-term value is the discovery of how controllable and/or regular stimulation induces a lasting beneficial effect that can prevent, and restore, adaptive plasticity and attenuate the development of EMR. We began to study this issue in collaboration with Gómez-Pinilla et al. (2007). Others had shown that LTP induces the expression of BDNF (Patterson et al., 1992), that mice with a BDNF deletion fail to exhibit LTP and that exogenous BDNF restores LTP (Patterson et al., 1996; Linnarsson et al., 1997). Other evidence indicated that BDNF can promote synaptic plasticity within the spinal cord. For example, intermittent hypoxia induces an adaptive modification within the cervical spinal cord known as phrenic long-term facilitation (Dale-Nagle et al., 2010). Local application of BDNF has a similar effect and the effect of intermittent hypoxia on neural function is blocked by a BDNF inhibitor (Baker-Herman et al., 2004). BDNF has also been shown to promote locomotor behavior after spinal injury (Boyce et al., 2007, 2012) and the beneficial effect of treadmill training on locomotor performance has been linked to an up-regulation of endogenous BDNF (Gómez-Pinilla et al., 2001). Finally, evidence suggests that TNF and BDNF impact synaptic scaling in opposite ways (Turrigiano, 2008).

**FIGURE 6:**
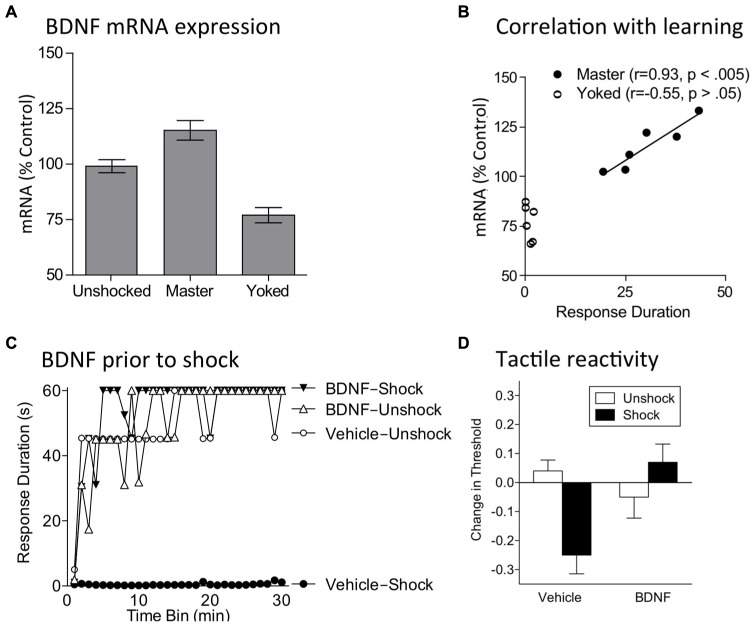
**BDNF mediates the beneficial effect of instrumental training. (A)** Training with controllable shock (Master) produces an increase in BDNF mRNA expression whereas exposure to uncontrollable shock (Yoked) down-regulates expression. **(B)** In trained subjects (Master), BDNF mRNA expression is highly correlated with a measure of learning (mean response duration during the first 10 min of training). **(C)** Subjects were given BDNF (i.t.; 0.4 μg) or its vehicle and then VIS (Shock) or nothing (Unshock). The next day subjects were tested in the instrumental learning paradigm. Prior exposure to shock impaired learning (Vehicle-Shock). Pretreatment with BDNF (BDNF-Shock) blocked the induction of this learning impairment. **(D)** Spinally transected rats received BDNF (i.t.; 0.4 μg), or its vehicle, followed by VIS (Shock) or nothing (Unshock). Tactile reactivity was tested bilaterally using von Frey stimuli applied to the plantar surface of each hind paw. Because similar results were observed across legs, the data were collapsed across this variable. Vehicle treated rats that received shock exhibited EMR. Pretreatment with BDNF blocked this effect. Adapted from Gómez-Pinilla et al. (2007) and Huie et al. (2012b).

Given these observations, we explored whether instrumental training affects BDNF expression in spinally transected rats. Subjects underwent training with controllable (Master) or uncontrollable (Yoked) shock and tissue was collected at the end of training. Relative to both unshocked and yoked groups, training with controllable stimulation up-regulated BDNF expression (**Figure 6A**; Gómez-Pinilla et al., 2007). In contrast, uncontrollable stimulation down-regulated expression. mRNA expression in master rats was well-correlated with an index of instrumental learning (**Figure 6B**). An identical pattern was observed for CaMKII and CREB mRNA expression. These genes were of interest because they are regulated by BDNF, have been implicated in other models of neural plasticity, and have been characterized as molecular memory switches (Yin et al., 1995; Yin and Tully, 1996; Blanquet and Lamour, 1997; Tully, 1997; Finkbeiner, 2000; Lisman et al., 2002). Using *in situ* hybridization, we showed that training with controllable shock induces an increase in BDNF mRNA expression within both the dorsal and ventral horn (Huie et al., 2012b). Western blotting showed BDNF protein was increased within the L3–L5 segments. Training also increased protein expression of the BDNF receptor TrkB [both truncated (TrkB 95) and full length (TrkB 145)]. Immunohistochemical analyses revealed increased TrkB protein expression within the dorsal horn and double labeling showed that most TrkB expression was localized to neurons.

Next, we assessed the impact of inhibiting BDNF function using the sequestering antibody TrkB-IgG. TrkB-IgG did not have a significant effect on instrumental learning (Gómez-Pinilla et al., 2007; Huie et al., 2012b). It did, however, block the facilitation of learning when subjects were tested at a higher response criterion (Gómez-Pinilla et al., 2007). Inhibiting the downstream signal CaMKII with AIP had the same effect. If training fosters learning because it up-regulates BDNF release, exogenous application of BDNF should promote learning. As predicted, intrathecal BDNF facilitated learning in untrained rats tested with a high response criterion (Gómez-Pinilla et al., 2007). This pattern of results implies that training induces a lasting modification that up-regulates BDNF expression, which promotes learning about new environmental relations and alters the capacity for future learning.

If controllable/predictable shock induces a protective effect because it up-regulates BDNF expression and release, then BDNF should substitute for training and block the induction of the VIS induced learning impairment. To test this, subjects received intrathecal BDNF followed by VIS. As usual, rats given VIS exhibited a learning impairment when tested 24 h later with controllable stimulation (Huie et al., 2012b). Pretreatment with BDNF blocked the induction of this learning deficit (**Figure 6C**).

As discussed above, the learning impairment observed after VIS can be eliminated by training rats with controllable stimulation [in conjunction with a drug (naltrexone) that blocks the expression of the learning deficit; Crown and Grau, 2001]. To examine whether this therapeutic effect of training depends upon BDNF, subjects were given VIS followed by instrumental training in the presence of naltrexone (Huie et al., 2012b). Prior to instrumental training, rats received TrkB-IgG or its vehicle. The next day, subjects were tested in our instrumental learning paradigm. As usual, training eliminated the VIS-induced learning impairment. This restorative effect was not observed in subjects given TrkB-IgG prior to instrumental training. Recognizing that TrkB-IgG could have blocked the beneficial effect of training, in part, by interfering with instrumental learning, we examined whether TrkB-IgG would be effective if given immediately after instrumental training. Again, rats received variable shock followed by instrumental training in compound with naltrexone. At the end of training, half the subjects received TrkB-IgG. We found that blocking BDNF *after* instrumental training attenuated its restorative effect. This suggests that the effect of TrkB-IgG is not due to a disruption of instrumental learning and implies that training induces a prolonged increase in BDNF release that contributes to the restoration of learning.

Having shown that BDNF is essential to the restorative effect of instrumental training, we asked whether exogenous BDNF could substitute for training and restore the capacity to learn in rats that had previously received VIS (Huie et al., 2012b). Subjects received VIS or nothing followed by intrathecal BDNF or vehicle. When tested in our instrumental paradigm 24 h later, subjects that had received VIS exhibited a learning impairment. BDNF given after VIS restored the capacity to learn. Further work revealed that BDNF given 24 h after VIS, immediately before testing, also eliminates the learning impairment.

VIS also induces EMR (Ferguson et al., 2006). Our results imply that BDNF may mediate this effect too. We examined this possibility by administering BDNF prior to 6 min of VIS applied to one hindlimb in spinally transected rats. Tactile reactivity was assessed using von Frey stimuli applied to the plantar surface of each hind paw. Our usual dose of BDNF (0.4 μg) had no effect on baseline tactile reactivity. Exposure to VIS induced EMR and this effect was blocked by BDNF (**Figure 6D**). More recently, we have shown that this same dose of BDNF counters inflammation-induced EMR in spinally transected rats and down-regulates a cellular marker of nociceptive sensitization (Erk phosphorylation; Lee et al., 2014).

In summary, we found that BDNF generally counters maladaptive plasticity, reinstating the capacity for learning and attenuating EMR in spinally transected rats. Our results further show that instrumental training and exposure to fixed spaced stimulation have a beneficial effect because they up-regulate BDNF expression (Gómez-Pinilla et al., 2007; Huie et al., 2012b). These finding complement other data demonstrating that locomotor training, exercise, and intermittent hypoxia, can promote adaptive plasticity through a BDNF-dependent process (Gómez-Pinilla et al., 2001; Baker-Herman et al., 2004).

How BDNF affects spinal function appears to be modulated by spinal injury. Specifically, in spinally injured rats BDNF attenuates EMR (Cejas et al., 2000; Huie et al., 2012b; Lee et al., 2014) whereas it often enhances pain in uninjured subjects (Merighi et al., 2008). As we discuss below, these differences may be related to the regulation of intracellular Cl^-^ concentrations, which can alter GABA function. Other important factors may include the BDNF source (neural or glial) and BDNF concentration (cf Miki et al., 2000; Cunha et al., 2010).

We found that training induced a rapid increase in BDNF protein, which was evident when tissue was collected immediately after 30 min of training. Likewise, intermittent hypoxia has been shown to increase BDNF protein within 60 min (Baker-Herman et al., 2004). These findings may reflect the local dendritic cleavage of the pro-form of BDNF into the mature form. This mechanism, which is mediated by tissue plasminogen activator (tPA), can be rapidly engaged in an activity-dependent manner (Waterhouse and Xu, 2009). Interestingly, in the absence of cleavage, pro-BDNF can have an opponent-like effect through its action at the P75 neurotrophin receptor (P75NTR; Bothwell, 1996; Lu et al., 2005; Cunha et al., 2010). For example, while BDNF fosters the development of LTP, proBDNF favors the induction of LTD. This suggests the intriguing possibility that training may influence BDNF function, in part, by regulating cleavage of proBDNF.

Our work suggests that BDNF plays a major role in mediating the restorative effect of behavioral training; that it is both necessary and sufficient to its expression. However, we have found no evidence that BDNF is required for the induction, or maintenance, of these training effects. Further, while BDNF can substitute for training to enable learning, and counter the adverse effect of uncontrollable/unpredictable stimulation, its effect appears to wane within a few hours (Zhang et al., 2014).

## METAPLASTICITY AND SPINAL CORD INJURY

### UNCONTROLLABLE STIMULATION IMPAIRS RECOVERY AND ENHANCES PAIN IN CONTUSED RATS

We have begun to explore the implications of our results for recovery after a contusion injury. Our work was motivated by both our studies in spinally transected rats and the clinical observation that spinal injuries are often accompanied by other tissue damage that provide a source of nociceptive input and peripheral inflammation. Our hypothesis was that afferent nociceptive signals could induce a state of over-excitation that enhances secondary damage and undermines recovery. Because we have a good understanding of how VIS induced nociceptive activity affects spinal function after a transection, and because this type of stimulation is readily controlled and produces (at the intensities used) no secondary peripheral effects that extend beyond the period of stimulation, we began by exploring the impact of VIS.

**FIGURE 7:**
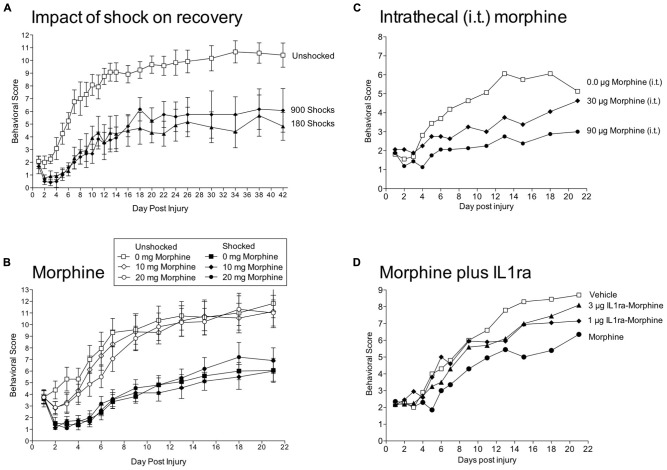
**Impact of shock and morphine treatment on recovery after a contusion injury. (A)** Rats received a moderate contusion injury and 24 h later VIS (180 or 900 shocks) or nothing (Unshocked). Locomotor recovery was scored over the next six weeks by experimenters that were blind to the subjects experimental treatment using the BBB scale (Basso et al., 1995). Scores were adjusted using the transformation derived by Ferguson et al. (2004), which improves the metric properties of the scale. Shock treatment impaired the recovery of locomotor function. **(B)** Contused rats were given morphine [0, 10, or 20 mg, intraperitoneal (i.p.)] followed by VIS (Shocked) or nothing (Unshocked). Behavioral observations confirmed that the highest dose of morphine blocked shock-elicited movement and vocalization. Morphine treatment did not block the adverse effect of shock treatment on locomotor recovery. **(C)** Intrathecal (i.t.) administration of morphine 24 h after a contusion injury produces a dose-dependent impairment in locomotor recovery. **(D)** A day after a contusion injury, rats were pretreated with the IL-1 receptor antagonist (IL1ra; 1 or 3 μg, i.t.) or its vehicle and then given morphine (90 μg, i.t.) or vehicle. Morphine alone impaired locomotor recovery and this effect was attenuated by pretreatment with IL1ra. Adapted from Grau et al. (2004) and Hook et al. (2008, 2007, 2009).

To assess the impact of stimulation on recovery, we used a moderate (12.5 mm) contusion injury at T12 produced with the MASCIS device (Grau et al., 2004). A day after injury, we assessed locomotor performance and then exposed rats to VIS. We found that VIS produced a profound disruption in recovery (**Figure 7A**). This effect was evident within 3 days and was maintained over the next 6 weeks. Further work showed that shock treatment was most effective when given within 4 days of injury. Most importantly, nociceptive stimulation only had an adverse effect on recovery when shock was given in an uncontrollable manner; subjects that received the same amount of shock, but could control its occurrence (by exhibiting a flexion response) exhibited normal recovery. Shock treatment also enhanced mortality, led to greater weight loss, slowed the recovery of bladder function, and led to a higher incidence of spasticity. Histological analyses revealed that uncontrollable intermittent shock enhanced tissue loss (white and gray matter) at the site of injury and increased damage caudal to injury (Grau et al., 2004; Hook et al., 2007).

More recently, we have examined whether VIS affects the development of EMR in contused subjects (Garraway et al., 2012). As others have reported, contused rats exhibited EMR relative to sham-operated subjects from 7 to 28 days after injury. Contused rats that received 6 min of VIS exhibited an EMR that emerged more rapidly (within 24 h of shock treatment) and remained more robust (7–28 days after injury).

### OPIOIDS DO NOT BLOCK THE EFFECT OF NOCICEPTIVE STIMULATION AND IMPAIR RECOVERY

Having shown that nociceptive stimulation impairs recovery after a contusion injury, we reasoned that inhibiting nociceptive transmission could have a protective effect. We first verified that an injection of morphine (20 mg/kg, i.p.) induced a robust antinociception on the tail-flick test in contused rats (Hook et al., 2007). Importantly, morphine also inhibited shock-elicit movements and brain-dependent responses to pain (e.g., vocalization). In morphine treated contused rats, VIS induced little movement or pain, but nonetheless impaired recovery (**Figure 7B**). Morphine not only failed to have a protective effect, it interacted with nociceptive stimulation and enhanced mortality. Indeed, half the subjects (8 out of 16) given both VIS and 20 mg/Kg of morphine died. Oddly, subjects typically died days after morphine treatment (mean = 4.6).

Systemic morphine could affect recovery by directly impacting a spinal process or by engaging a brain system that indirectly affects spinal function. We hypothesized that the drug effect was due to a direct mode of action. To show this, we tested the impact of intrathecal morphine given 24 h after a contusion injury (Hook et al., 2009). Again, we confirmed that drug treatment induced a robust antinociception. Intrathecal morphine (90 μg) impaired the recovery of locomotor function (**Figure 7C**), led to greater weight loss, increased tissue loss at the site of injury, and enhanced rear paw-directed grooming/chewing (autophagia), a potential index of neuropathic pain.

Morphine has been shown to up-regulate proinflammatory cytokines [e.g., interleukin-1β (IL-1β), interleukin-6 (IL-6), TNF; Song and Zhao, 2001; Johnston et al., 2004]. Consistent with this work, systemic morphine (20 mg/kg) a day after a contusion injury increased expression of IL-1β and IL-6 24 h after drug treatment (Hook et al., 2011). Intrathecal morphine had a similar effect and increased IL-1β within 30 min of drug treatment. To explore whether the release of IL-1β was causally related to the adverse effect of morphine treatment, we administered a IL-1 receptor antagonist (IL-1ra) prior to intrathecal morphine (90 μg). Morphine impaired locomotor recovery and this effect was blocked by IL-1ra (**Figure 7D**). Three weeks after injury, morphine treated rats also showed increased vocalization to tactile stimulation applied to the girdle region, an indication of increased at-level pain. This effect too was blocked by IL-1ra. While these results are promising, we also found that IL-1ra treatment led to greater issue loss at the site of injury, presumably because it blocked a beneficial effect of injury-induced IL-1β expression.

Current research is exploring the site of opioid action. As described above, the kappa-2 agonist GR89696 inhibits adaptive plasticity in transected rats. This same drug also impairs recovery after a contusion injury (Aceves and Hook, 2013). This is consistent with early studies that linked contusion-induced damage to kappa opioid activity (Faden, 1990). Other work suggests that opioids can also engage glia, and promote cytokine release, by engaging non-classic receptors [e.g., the toll-like receptor 4 (TLR4); Hutchinson et al., 2007, 2010; Watkins et al., 2007]. It seems likely that the adverse effects of morphine on spinal function are due to its action at multiple sites, including TLR4.

While morphine did not block the adverse effect of nociceptive stimulation, the data yielded an important discovery—opioid treatment after a contusion injury impairs the recovery of locomotor function, enhances pain, and leads to greater tissue loss (Hook et al., 2007, 2009). Further, when combined with nociceptive stimulation, morphine enhanced mortality. These results are especially troubling given the widespread use of opioids to treat pain after SCI (Warms et al., 2002; Widerstrom-Noga and Turk, 2003).

The results also have implications regarding the mechanisms that underlie the adverse effect of VIS on recovery. For example, it could be argued that this effect is secondary to brain-mediated pain or VIS-induced movement. Morphine treatment blocked both behavioral signs of pain and VIS-induced movement, but did not attenuate the effect of VIS on recovery. Further, if brain systems exert a protective effect by inhibiting spinal nociceptive transmission, our results imply that this antinociception is mediated by a nonopioid process (Meagher et al., 1993). Finally, the data indicate that a kappa-2 opioid dependent process, that we have shown inhibits adaptive plasticity in transected rats (Washburn et al., 2008), can substitute for VIS treatment (i.e., is sufficient) and impair recovery after a contusion injury (Aceves and Hook, 2013).

### UNCONTROLLABLE STIMULATION INCREASES TNF AND REDUCES BDNF IN CONTUSED RATS

Earlier we described how the learning impairment induced by VIS in transected rats depends upon TNF (Huie et al., 2012a). Given this, we examined whether VIS induces TNF expression in contused subjects (Garraway et al., 2012). We found that nociceptive stimulation a day after a contusion injury increased TNF mRNA and protein expression from 1 to 7 days after VIS treatment. Interestingly, stimulation also increased protein levels of caspase 3 and 8, two indices of programmed cell death (apoptosis; Beattie et al., 2000; Duprez et al., 2009). Immunofluorescent labeling revealed that caspase 3 was co-labeled with OX-42 (microglia) and NeuN (neurons), but not GFAP (astrocytes). These observations parallel the results found with the transection paradigm and suggest that TNF release may foster secondary damage by promoting apoptotic cell death.

**FIGURE 8:**
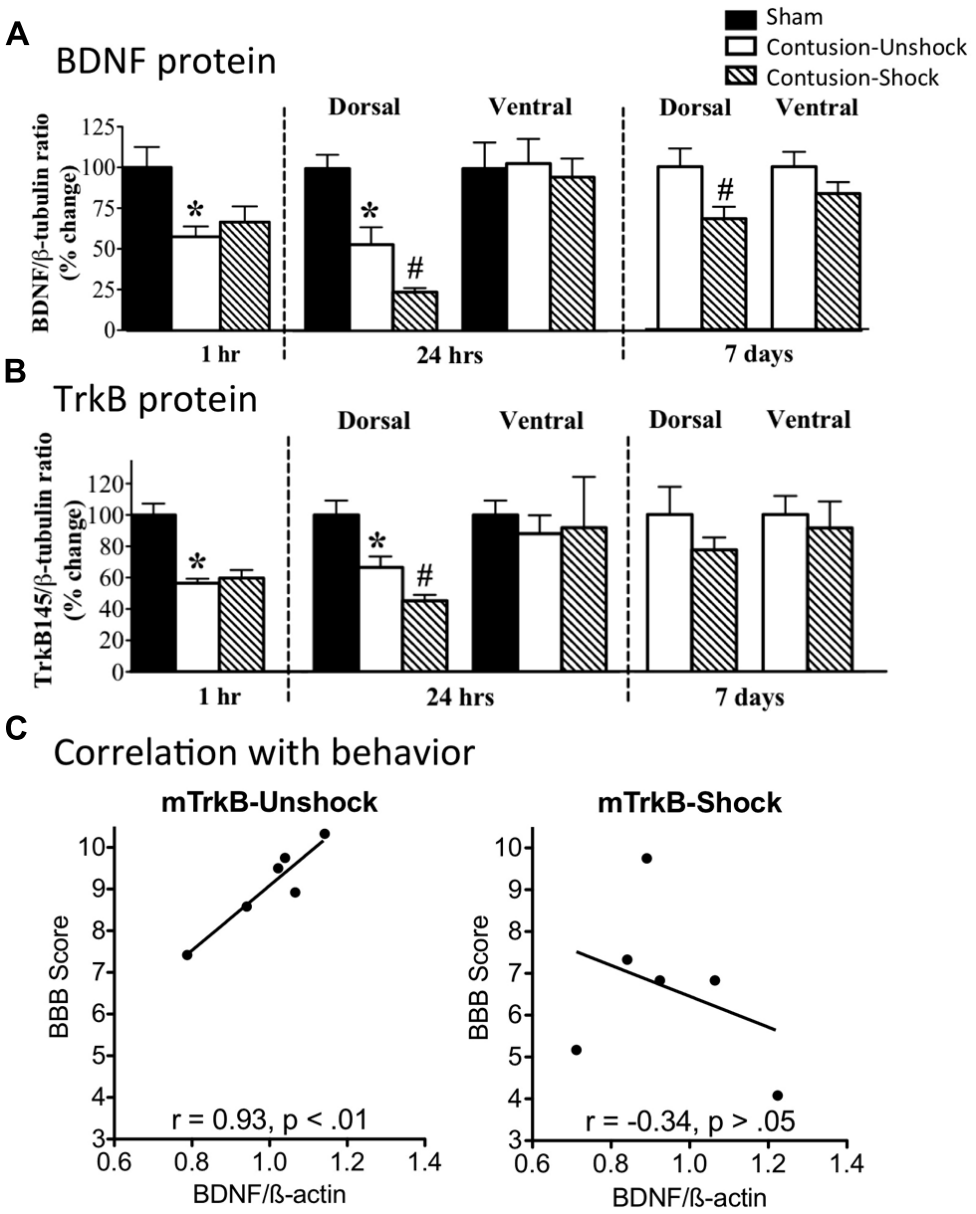
**Impact of VIS on BDNF and TrkB expression in contused rats. (A)** Rats received a moderate contusion injury or a sham surgery. The next day, contused rats received VIS (Contused-Shock) or nothing (Contused-Unshock). BDNF protein was assayed 1 h, 24 h, and 7 days after treatment. A contusion injury produced a significant decrease (*) in BDNF expression at 1 and 24 h (25 and 48 h after surgery). Shock treatment further down-regulated BDNF expression (#) at 24 h and 7 days. **(B)** TrkB protein expression was also down regulated by a contusion injury at 1 and 24 h (*). Shock treatment produced a further decrease at 24 h (#). **(C)** Locomotor performance on days 2–7 was highly correlated with mTRKB expression in untreated (Unshock) contused rats (left panel), but not in rats that received shock (right panel). Adapted from Garraway et al. (2011).

We have also examined the impact of nociceptive stimulation on BDNF/TrkB expression after a contusion injury (Garraway et al., 2011). Our hypothesis was that uncontrollable nociceptive stimulation impairs recovery, in part, by down-regulating BDNF expression. To test this, subjects were given a moderate contusion injury and were exposed to VIS or nothing the next day. A contusion injury, *per se*, down-regulated BDNF mRNA and protein expression (**Figure 8A**). Exposure to VIS a day after injury further down-regulated BDNF mRNA and protein expression and this effect was most evident a day after shock treatment (48 h after injury). In the dorsal horn, VIS induced a lasting reduction in BDNF protein that was evident a week after shock treatment. A contusion injury and VIS treatment had a similar effect on TrkB mRNA and protein expression (**Figure 8B**), reducing expression during the first 48 h of recovery. TrkB immunolabeling showed that it was co-expressed with NeuN, but not GFAP or OX-42. Correlational analyses revealed that locomotor recovery was highly related with TrkB mRNA expression in unshocked, but not shocked, subjects (**Figure 8C**). A similar pattern was observed for BDNF. This suggests that improved recovery is normally associated with enhanced TrkB expression and that shock treatment may adversely affect recovery by dysregulating this process.

In summary, the results obtained to date generally parallel the findings obtained in our transection paradigm. In both cases, exposure to VIS has a maladaptive effect that induces EMR and disrupts adaptive plasticity, impairing both instrumental learning and recovery after a contusion injury (Grau et al., 1998, 2004; Joynes et al., 2003; Ferguson et al., 2008; Garraway et al., 2011, 2012). As observed in transected rats, VIS induces an increase in TNF expression and down-regulates BDNF in contused subjects (Gómez-Pinilla et al., 2007; Garraway et al., 2011, 2012; Huie et al., 2012a). The contusion paradigm also showed that nociceptive stimulation engages markers of apoptotic cell death and leads to enhanced tissue loss. These adverse effects may explain, in part, why other types of nociceptive stimulation (e.g., from stretching; Caudle et al., 2011) impair the recovery process.

## SPINAL PROCESSES ARE REGULATED BY THE BRAIN

### ANESTHESIA BLOCKS THE BRAIN-DEPENDENT INHIBITION OF MALADAPTIVE PLASTICITY

**FIGURE 9:**
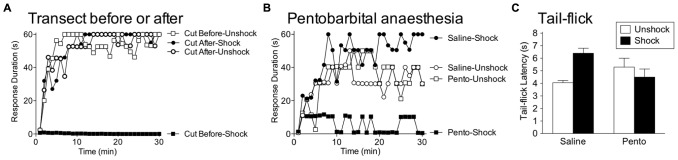
**Brain systems transform how shock affects spinal function. (A)** Rats received VIS (Shock) or nothing (Unshock) before or after a spinal transection (Cut) and were tested for instrumental learning the next day. When the spinal cord was cut prior to treatment, shock induced a learning impairment (Cut Before-Shock). When the spinal cord was cut after shock treatment (Cut After-Shock), no impairment was observed. **(B)** Intact rats were anesthetized with pentobarbital (50 mg/kg, i.p.) or given saline. Subjects then received VIS (Shock) or nothing (Unshock), followed by a spinal transection and instrumental testing. Shock induced a learning impairment in anesthetized (Pento-Shock) but not awake (Saline-Shock) rats. **(C)** Intact rats received saline or pentobarbital (50 mg/kg, i.p.) followed by VIS (Shock) or nothing (Unshock). Nociceptive reactivity was tested by applying a noxious thermal stimulus to the tail and measuring the latency to exhibit a tail-withdrawal (tail-flick). In awake rats (Saline), shock treatment induced antinociception. Shock had no effect on tail-flick latencies in anesthetized (Pento) rats. Adapted from Crown and Grau (2005) and Washburn et al. (2007).

We have shown that VIS inhibits spinal plasticity in transected animals and impairs recovery after a contusion injury (Grau et al., 1998, 2004). Does this effect impact spinal function in the absence of injury? The answer appears to be a qualified no. We explored this issue by applying VIS before or after a spinal transection (Crown and Grau, 2005). The next day we tested subjects in our instrumental paradigm. As usual, VIS given after a spinal transection induced a learning impairment (**Figure 9A**). When given before, it had no effect, which suggests that brain-dependent processes exert a modulatory effect that counters the development of the learning impairment. This is consistent with other studies showing that the induction of spinal LTP is inhibited by descending pathways (Sandkühler and Liu, 1998; Sandkühler, 2000). The results suggest that the brain normally acts to quell over-excitation within the spinal cord and thereby helps to maintain neural homeostasis. We would also expect this process to counter the development of central sensitization. Supporting this, we recently found that capsaicin-induced EMR is weaker in intact subjects (relative to spinally transected; Huang et al., 2014).

A caveat to our description of brain-dependent regulation is that the protective role of brain processes can be disrupted by surgical anesthesia. This issue was explored by Washburn et al. (2007), who tested whether VIS induced a learning impairment in pentobarbital anesthetized rats. Others have suggested that pentobarbital anesthesia induces a physiological state within the spinal cord that resembles the consequences of a spinal transection (Mori et al., 1981). Given this, she predicted that VIS applied to the tail would have its usual effect in intact anesthetized rats. To test this, intact subjects received anesthetic dose of pentobarbital or its vehicle followed by VIS. The subjects were then transected and a day later, tested for instrumental learning. As expected, VIS did not induce a learning impairment in unanesthetized subjects (**Figure 9B**). Rats that received pentobarbital prior to VIS failed to learn, implying that pentobarbital anesthesia eliminates the brain-dependent protection of spinal circuitry. This finding is important because it suggests that nociceptive input during general anesthesia could have an unanticipated effect that sensitizes spinal nociceptive systems and promotes the development of neuropathic pain.

As noted earlier, VIS does not induce antinociception in spinally transected rats. However, in intact rats, VIS induces a robust antinociception (**Figure 9C**). This effect too was blocked by pentobarbital anesthesia (Washburn et al., 2007). The finding implies that brain-dependent processes may protect spinal systems by inhibiting nociceptive transmission. Because pretreatment with an opioid does not have a protective effect (Hook and Grau, 2007), we posit that brain systems inhibit the development of central sensitization through a non-opioid process (e.g., serotonin).

### DESCENDING SEROTONERGIC FIBERS MEDIATE THE INHIBITION OF MALADAPTIVE PLASTICITY

**FIGURE 10:**
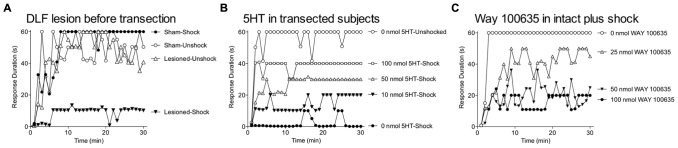
**Descending serotonergic systems modulate the induction of the shock-induced learning impairment. (A)** Subjects underwent bilateral lesions of the DLF at T2 or a sham surgery. Two hours later the spinal cord was transected at T8 and instrumental learning was assessed the next day. Shock treatment induced a learning impairment in DLF lesioned rats (Lesioned-Shock) but not in intact sham operated rats (Sham-Shock). **(B)** Spinally transected rats received serotonin (i.t.; 0, 10, 50, or 100 nmol) followed by VIS (Shock) or nothing (Unshock). Instrumental learning was tested the next day. Shock treatment impaired learning in vehicle treated rats (0 nmol 5HT-Shock). This learning impairment was blocked by pretreatment with 5HT in a dose-dependent manner. **(C)** Intact rats received the 5HT 1A antagonist WAY 100635 (i.t.; 0, 25, 50, or 100 nmol) prior to VIS. The spinal cord was then transected and instrumental learning was assessed the next day. As expected, intact rats that received shock and the vehicle (0 nmol WAY 100635) did not exhibit a learning impairment. Shock induced a learning impairment in subjects given the 5HT 1A antagonist and this effect was dose-dependent. Adapted from Crown and Grau (2005).

Prior work suggested that the inhibition of the VIS-induced learning impairment could be mediated by serotonergic fibers that descend through the dorsal lateral funiculus (DLF; Davies et al., 1983; Watkins et al., 1984). If this is true, we should be able to eliminate the brain-dependent protection of spinal circuits by lesioning the DLF. Crown and Grau (2005) examined this issue by bilaterally lesioning the DLF at T2. Relative to the sham operated controls, DLF lesions had little effect on sensory/motor function. Subjects then received VIS and 2 h later the spinal cord was transected at T8. The capacity for instrumental learning was assessed the next day. As expected, in the absence of DLF lesions, VIS had no effect on spinal function. However, in DLF lesioned rats VIS induced a learning impairment (**Figure 10A**).

Because a large proportion of the fibers within the DLF are serotonergic (Davies et al., 1983), we hypothesized that the brain-mediated protective effect depends upon serotonin (5HT). This is consistent with other work that has linked the development of EMR after spinal injury to the loss of 5HT. Further, 5HT has been shown to attenuate EMR in injured rats and this effect has been linked to an action at the 5HT 1A receptor (e.g., Eaton et al., 1997; Bardin et al., 2000; Gjerstad et al., 2001; Hains et al., 2001a,b, 2003). If this same system mediates the brain-dependent inhibition of the learning impairment, engaging the 5HT-1A receptor should have a protective effect in spinally transected rats. To test this, rats were spinally transected and administered the agonist 5HT or 8-OH DPAT (5HT 1A/7) intrathecal prior to VIS. Instrumental learning was tested the next day. Crown and Grau (2005) found that both drugs block the VIS-induced learning impairment (**Figure 10B**). Drugs that acted at other 5HT receptors [DOI (5HT 2) and quipazine (5HT 2/3)] had no effect. Recognizing that some fibers within the DLF are noradrenergic (Davies et al., 1983), Crown and Grau (2005) also assessed the impact of the α-2 noradrenergic agonist clonidine. While clonidine had a protective effect, its action was blocked by a 5HT 1A antagonist (WAY 100635), implying that the positive effect was due to cross-reactivity with the 5HT 1A receptor (Newman-Tancredi et al., 1998; Shannon and Lutz, 2000).

These results suggest that activation of the 5HT 1A receptor can substitute for the brain-dependent process in transected rats to inhibit the development of maladaptive plasticity. To test the necessity of this process, Crown blocked the 5HT 1A receptor in intact rats (using i.t. WAY 100635) and administered VIS. The spinal cord was then transected and subjects were tested for instrumental learning. When the 5HT 1A receptor was blocked, VIS induced a learning impairment in intact rats (**Figure 10C**). We conclude that descending serotonergic fibers counter the development of maladaptive plasticity by engaging the 5HT 1A receptor. The corollary to this is that damage to this tract will remove this protective effect and set the stage for maladaptive plasticity in response to uncontrollable nociceptive input and peripheral inflammation.

### SPINAL INJURY ALTERS GABAERGIC FUNCTION WITHIN THE SPINAL CORD

Our results imply that the loss of brain input can fundamentally alter how spinal systems operate. We suggest that this may explain why some treatments affect spinal systems in opposite ways depending whether brain input is intact or absent. An especially revealing example of this emerged from our work examining the role of GABAergic systems (Ferguson et al., 2003). We had shown that administration of the GABA-A antagonist bicuculline blocks the expression of the VIS-induced learning impairment. VIS also induces EMR (Ferguson et al., 2006). If the learning impairment and the EMR are mediated by a common mechanism, the EMR should also be blocked by bicuculline. This seemed paradoxical because GABA is typically viewed as having an inhibitory effect that should counter neural excitation and EMR. Indeed, in intact rats, blocking GABA with bicuculline generally enhances reactivity to nociceptive and mechanical stimuli (Roberts et al., 1986; Millan, 2002). However, in spinally transected rats, we found that intrathecal bicuculline blocks VIS-induced EMR (Huang et al., 2011). More surprising, treatment with bicuculline also blocked capsaicin-induced EMR, and cellular indices of central sensitization (e.g., ERK phosphorylation), in transected rats (Huang et al., 2014). Thus, when communication with the brain was disrupted, blocking GABAergic activity seemingly quelled, rather than enhanced, nociceptive sensitization.

**FIGURE 11:**
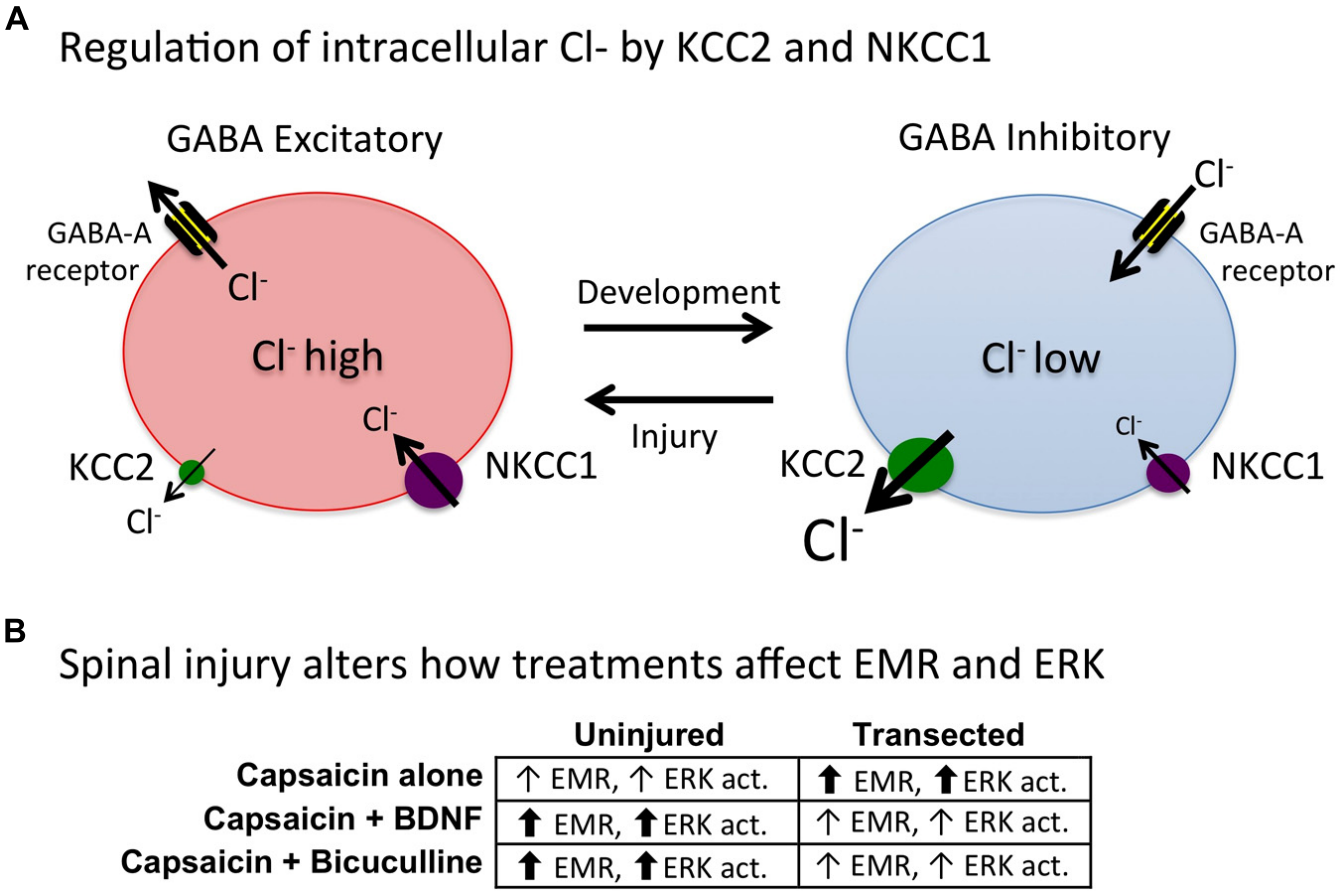
**Injury increases intracellular Cl^-^ in GABA-responsive cells, causing GABA to have an excitatory effect and transforming how experimental manipulations impact spinal function. (A)** Schematic of the processes that regulate intracellular Cl^-^. NKCC1 regulates the inward flow of Cl^-^ whereas KCC2 controls its outward flow. Early in development, membrane-bound (plasmalemmal) KCC2 levels are low, and as result, the intracellular concentration of Cl^-^ remains high. Under these conditions, engaging the GABA-A receptor allows Cl^-^ to exit the cell, causing it depolarize, which promotes neural excitation. With development, there is an up-regulation of KCC2, which drives down the intracellular concentration of Cl^-^. This causes a shift in polarity because engaging the GABA-A receptor now causes Cl^-^ to flow into the cell, inducing hyperpolization and neural inhibition. Recent work has revealed that spinal injury can cause a regression to the immature state, by down-regulating KCC2 expression (Ben-Ari et al., 2012). This would reduce the hyperpolarizing effect of GABA and enhance the degree to which it induces neural excitation. There is also a complimentary change in NKCC1 function, which is regulated through phosphorylation (Flemmer et al., 2002). **(B)** Capsaicin produces a stronger EMR, and greater ERK activation, in spinally transected rats. After a spinal injury, pretreatment with BDNF or bicuculline attenuates capsaicin-induced EMR and ERK activation. In uninjured rats, BDNF and bicuculline enhance capsaicin-induced EMR and ERK activation. Adapted from Kahle et al. (2014).

The fault in our reasoning likely lies with the assumption that GABA uniformly has an inhibitory effect. The impact of GABA on neural excitation is regulated by the concentration of intracellular Cl^-^, which is controlled by K^+^-Cl^-^ cotransporter 2 (KCC2) and Na^+^-K^+^-Cl^-^ cotransporter 1 (NKCC1; **Figure 11A**). As the nervous system develops, there is an increase in KCC2 expression, which lowers the concentration of intracellular Cl^-^ (Ben-Ari, 2002). As a result, engaging the GABA-A receptor causes Cl^-^ to flow into the cell, which produces neural inhibition. Spinal injury, however, can cause a reduction in KCC2 expression, which leads to an increase in intracellular Cl^-^ (Ben-Ari et al., 2012). Consequently, engaging the GABA-A receptor will allow Cl^-^ to flow out of the cell, which has a depolarizing (excitatory) effect that may contribute to the development of EMR (Cramer et al., 2008; Hasbargen et al., 2010). Recently, we confirmed that a spinal transection reduces the ratio of membrane bound KCC2 (relative to the cytosolic fraction) in the lumbosacral spinal cord within 24 h (Huang et al., 2014).

In the uninjured system, GABAergic transmission would have a homeostatic effect that would act to counter nociception-induced over-excitation. But if KCC2 levels are low, engaging this process would promote over-excitation and the development of central sensitization. Under these conditions, pretreatment with a GABA-A antagonist should counter the development of central sensitization and EMR, and our data suggest that this is true (Huang et al., 2013). We would further predict that in intact animals, intrathecal bicuculline should have the opposite effect. In the intact system, plasmalemmal KCC2 levels should be high. Now, engaging the GABA-A receptor would promote the inward flow of Cl^-^, producing neural inhibition. Supporting this, we found that in intact animals, bicuculline enhanced capsaicin-induced EMR and ERK phosphorylation (Huang et al., 2014).

Changes in GABAergic function may also help to explain another paradoxical effect. Earlier, we described how intrathecal administration of BDNF has a beneficial effect in spinally transected rats, countering both the learning impairment and EMR induced by VIS (Huie et al., 2012b). More recently, we showed that BDNF also attenuates capsaicin-induced EMR in transected subjects (Lee et al., 2012). These effects on EMR run counter to other studies demonstrating that BDNF in intact subjects can foster the development of central sensitization and enhance pain (Merighi et al., 2008). In intact subjects, BDNF reduces plasmalemmal KCC2 within the lumbosacral spinal cord, which would reduce GABAergic inhibition and explain, in part, why BDNF promotes the development of central sensitization (Coull et al., 2005; Valencia-de Ita et al., 2006; Lu et al., 2009; Biggs et al., 2010). Interestingly, after spinal injury, BDNF appears to have the opposite effect on KCC2 expression (Boulenguez et al., 2010), which would re-establish the inhibitory action of GABA. This suggests that BDNF should attenuate inflammation-induced EMR in transected rats, which is what we observed (Lee et al., 2012). It further suggests that BDNF should have the opposite effect on capsaicin-induced EMR in intact rats. Lee et al. (2014) recently confirmed that this too is true using both behavioral and cellular indices of central sensitization.

In summary, our results suggest that spinal injury removes a brain-dependent protective effect that is mediated by descending serotonergic fibers and the 5HT-1A receptor. A loss of brain communication also leads to a shift in KCC2 that transforms how GABA affects spinal circuits, causing it to have an excitatory effect that we suggest contributes to the development of central sensitization. BDNF may have a protective effect, in part, by promoting KCC2 plasmalemmal expression. This would re-establish GABA-mediated inhibition (Boulenguez et al., 2010), which could counter the development of neural excitation. In the absence of injury, GABAergic inhibition would be blocked by bicuculline and reduced by BDNF (through a down-regulation of KCC2), and in both cases, this would promote nociceptive activity and the development of EMR (**Figure 11B**). These conclusions are consistent with electrophysiological data indicating that BDNF facilitates AMPA and NMDA mediated currents in intact, but not spinally transected, subjects (Garraway et al., 2003, 2005; Garraway and Mendell, 2007). These data suggest that BDNF is pronociceptive in intact subjects, but not after spinal injury.

## CONCLUSION

Studies of brain plasticity have uncovered processes that have a lasting impact on plastic potential, and we have suggested that this concept of metaplasticity has relevance to spinal function. We have shown that uncontrollable/unpredictable stimulation, and peripheral inflammation, induce a process that has a lasting inhibitory effect (Grau et al., 1998; Hook et al., 2008; Baumbauer et al., 2009). We related this process to the development of central sensitization and EMR, and characterized these effects as examples of maladaptive plasticity. We showed that predictable/controllable stimulation engages an opponent process, that fosters a form of adaptive plasticity (instrumental learning) and that counters the adverse effects of VIS and peripheral inflammation (Crown and Grau, 2001; Crown et al., 2002a). The beneficial effects of training appear related to an up-regulation of BDNF (Huie et al., 2012b). The adverse effects were tied to multiple processes: The expression of the deficit depends on kappa opioid activity (Washburn et al., 2008); its induction is coupled to the mGluR, non-neuronal cells, and the cytokine TNF (Ferguson et al., 2008; Vichaya et al., 2009; Huie et al., 2012a). We also showed that VIS disrupts recovery after a contusion injury and that this adverse effect was related to an up-regulation of TNF and a down-regulation of BDNF (Garraway et al., 2011, 2012).

Spinal injury appears to set the stage for damage and EMR by causing a loss of serotonergic fibers, which in the uninjured system, counter the development of maladaptive plasticity by acting at the 5HT-1A receptor (Crown and Grau, 2005). Disconnected from the brain, GABAergic systems revert to an immature state, due to a down-regulation of plasmalemmal KCC2 (Hasbargen et al., 2010; Ben-Ari et al., 2012). This causes GABA to have an excitatory effect that we posit contributes to the development of maladaptive plasticity.

We have linked the learning impairment to the release of TNF from microglia. The beneficial effects of training were tied to the release of BDNF. While we have not identified the relevant source of BDNF, it too could be expressed by microglia. If so, activity within microglia would determine whether an earlier experience engages a metaplastic effect that promotes (BDNF) or interferes (TNF) with adaptive plasticity. A potentially more intriguing question is whether microglia support a kind of biological switch that maintains enhanced expression. Such a process could be initiated by the profile of extracellular signals secreted by neurons during training. For example, the relative release of adenosine triphosphate (ATP), glutamate, and matrix metalloprotein-9 (MMP9), may vary depending upon whether the afferent signals are predictable/controllable versus unpredictable/uncontrollable. This could initiate alternative processes in microglia and astrocytes that are preserved over time and relayed to remote sites (Hansen et al., 2013).

It has long been recognized that the core (primary) injury affects the surrounding tissue to promote tissue loss (secondary injury; Beattie and Bresnahan, 2000). Our work shows that how this process unfolds is affected by peripheral stimulation. This is clinically important because SCI is often accompanied by other tissue damage (polytrauma; Chu et al., 2009; Putz et al., 2011), which provides a source of nociceptive input that can fuel nociceptive sensitization and promote cell death. Such a view anticipates that inhibiting neural excitation (e.g., by local cooling or administration of a Na^++^ channel blocker), or microglia activation (e.g., using minocycline), should reduce secondary damage and attenuate chronic pain (Baptiste and Fehlings, 2006; Batchelor et al., 2013; Hansebout and Hansebout, 2014).

In considering alternative treatments, the focus is on spinally mediated nociception, not conscious pain. Surgical anesthesia blocks the experience of pain, but does not protect spinal circuits. Rather, it allows nociceptive stimulation to induce a maladaptive effect in the absence of spinal injury (Washburn et al., 2007). Likewise, systemic morphine eliminates behavioral signs of pain, but does not counter the adverse effect peripheral stimulation has on spinal function and, worst yet, increases the extent of secondary injury (Hook et al., 2007, 2009).

Peripheral stimulation can also have an adverse effect during the chronic phase of injury, by inducing a form of maladaptive plasticity that inhibits learning and promotes nociceptive sensitization. Potential sources of nociceptive input include peripheral inflammation (e.g., from bed sores), stretching (Caudle et al., 2011), and electrical stimulation to induce muscle activity (Creasey et al., 2004). While pharmacological treatments that attenuate neural excitation can lessen the development of maladaptive plasticity, they will also inhibit adaptive plasticity and undermine the effectiveness of physical therapy. A better approach may involve training with predictable/controllable forms of stimulation, because these should both inhibit nociceptive sensitization and promote adaptive plasticity. Further, the link to metaplasticity implies that effective training can have a long-term benefit. Behavioral control is also relevant to the design of robotic systems (e.g., Del-Ama et al., 2014; McGie et al., 2014). These observations fit well with studies demonstrating long-term benefits of locomotor training (Edgerton et al., 2004). The work also implies that encouraging active behavioral control can enhance the beneficial effects of treatments that enable spinal function [e.g., epidural stimulation (Harkema et al., 2011; Angeli et al., 2014)].

## Conflict of Interest Statement

The Guest Associate Editor Shawn Hochman declares that, despite being affiliated to the same institution as author Sandra M. Garraway, the review process was handled objectively and no conflict of interest exists. The authors declare that the research was conducted in the absence of any commercial or financial relationships that could be construed as a potential conflict of interest.
